# Targeting aldolase A in hepatocellular carcinoma leads to imbalanced glycolysis and energy stress due to uncontrolled FBP accumulation

**DOI:** 10.1038/s42255-024-01201-w

**Published:** 2025-01-20

**Authors:** Marteinn T. Snaebjornsson, Philipp Poeller, Daria Komkova, Florian Röhrig, Lisa Schlicker, Alina M. Winkelkotte, Adriano B. Chaves-Filho, Kamal M. Al-Shami, Carolina Dehesa Caballero, Ioanna Koltsaki, Felix C. E. Vogel, Roberto Carlos Frias-Soler, Ramona Rudalska, Jessica D. Schwarz, Elmar Wolf, Daniel Dauch, Ralf Steuer, Almut Schulze

**Affiliations:** 1 Division of Tumor Metabolism and Microenvironment, German Cancer Research Center (DKFZ), Heidelberg, Germany; 2 Faculty of Biosciences, Heidelberg University, Heidelberg, Germany; 3 Institute for Theoretical Biology, Humboldt University of Berlin, Berlin, Germany; 4 Department of Biochemistry and Molecular Biology, Theodor-Boveri-Institute, University of Würzburg, Würzburg, Germany; 5 Department of Medical Oncology and Pneumology, University Hospital Tübingen, Tübingen, Germany; 6 IFIT Cluster of Excellence EXC 2180 ‘Image-Guided and Functionally Instructed Tumor Therapies’, University of Tübingen, Tübingen, Germany; 7 Biochemical Institute, University of Kiel, Kiel, Germany; 8 Tübingen Center for Academic Drug Discovery & Development (TüCAD2), Tübingen, Germany; 9 Peter Debye Institute for Soft Matter Physics, Leipzig University, Leipzig, Germany

**Keywords:** Cancer metabolism, Metabolomics, Cell growth, Hepatocellular carcinoma, Metabolism

## Abstract

Increased glycolytic flux is a hallmark of cancer; however, an increasing body of evidence indicates that glycolytic ATP production may be dispensable in cancer, as metabolic plasticity allows cancer cells to readily adapt to disruption of glycolysis by increasing ATP production via oxidative phosphorylation. Using functional genomic screening, we show here that liver cancer cells show a unique sensitivity toward aldolase A (ALDOA) depletion. Targeting glycolysis by disrupting the catalytic activity of ALDOA led to severe energy stress and cell cycle arrest in murine and human hepatocellular carcinoma cell lines. With a combination of metabolic flux analysis, metabolomics, stable-isotope tracing and mathematical modelling, we demonstrate that inhibiting ALDOA induced a state of imbalanced glycolysis in which the investment phase outpaced the payoff phase. Targeting ALDOA effectively converted glycolysis from an energy producing into an energy-consuming process. Moreover, we found that depletion of ALDOA extended survival and reduced cancer cell proliferation in an animal model of hepatocellular carcinoma. Thus, our findings indicate that induction of imbalanced glycolysis by targeting ALDOA presents a unique opportunity to overcome the inherent metabolic plasticity of cancer cells.

## Abstract (web summary)

Snaebjornsson et al. explore a selective vulnerability in cancer elicited by targeting aldolase A, which causes energy imbalance and prevents further metabolic adaptations.

## Main

Increased glucose uptake and aerobic glycolysis are among the central features of metabolic reprogramming of cancer cells^1^; however, in contrast to the historical view of the ‘Warburg effect’, it is now well recognized that most cancer cells also rely on oxidative metabolism in the mitochondria, both to produce energy as well as to generate various biosynthetic precursors derived from the tricarboxylic acid (TCA) cycle^2^. The high dependency of cancer cells on glucose uptake is therefore likely caused by the need for biomass production via pathways that branch off from glycolysis, such as the pentose phosphate pathway (PPP), the hexosamine pathway and serine synthesis pathway, rather than energy generation per se^3^. Indeed, it has been shown that various cancer cells are able to proliferate in the complete absence of glucose when supplemented with uridine, which can be catabolized to ribose-5-phosphate (R5P) and enter the PPP^4–6^. The conversion of fructose 6-phosphate (F6P) to fructose 1,6-bisphosphate (FBP) by the enzyme phosphofructokinase 1 (PFK1) is one of the rate-limiting steps in glycolysis and therefore tightly regulated^7^. PFK1 is generally highly active in cancer cells due to allosteric activation^8^, but modulation of its activity, for example by the TP53-induced glycolysis and apoptosis regulator (TIGAR)^9^ or the 6-phosphofructo-2-kinase/fructose-2,6-biphosphatase 4 (PFKFB4)^10^ can also promote cancer cell viability by supporting flux into the PPP. Furthermore, synthesis of FBP by PFK1 represents the last step of the investment phase and the beginning of the payoff phase of glycolysis in which the breakdown of FBP to pyruvate results in the net yield of two ATP molecules per molecule of glucose. Notably, the steady-state level of FBP has been identified as an intracellular indicator for glycolytic flux in a wide variety of systems, such as prokaryotes^11^, yeasts^12,13^, mouse embryos^14^ and cancer cells^7^.

Glycolysis is an autocatalytic pathway and carries an inherent risk of the investment phase outpacing the payoff phase, a state termed ‘imbalanced glycolysis’, which leads to a rapid depletion of intracellular ATP^15,16^ and subsequent cell cycle arrest or cell death. In yeast this has been observed upon the loss of the enzyme trehalose 6-phosphate (T6P) synthetase (TPS1), which catalyses the first ATP-dependent step in the conversion of glucose-6-phosphate to trehalose, with the product of this reaction T6P functioning as an allosteric inhibitor of hexokinase (HK)^17^. Mammalian cells lack the trehalose pathway and it is the glycolytic intermediate glucose 6-phosphate (G6P) which regulates HK activity by inhibiting the enzyme. Indeed, overexpression of glucokinase in insulinoma cells^18^ and HK2 in iBMK cells was shown to induce rapid ATP depletion^7^ reminiscent of imbalanced glycolysis. Glucokinase is insensitive to G6P inhibition and HK2, which is upregulated in various cancers^19^, has been shown to alleviate this inhibition by interacting with voltage-dependent anion channels at the mitochondrial outer membrane^20–23^. It is thus clear that the allosteric regulation of hexokinase via the trehalose pathway in yeast and via G6P in mammals has evolved as a natural safeguard mechanism against the formation of imbalanced glycolysis. Moreover, as high glucose uptake and elevated glycolytic flux are unifying features of cancers due to the constitutive activation of upstream signalling pathways, inducing imbalanced glycolysis could represent an attractive therapeutic strategy.

Here we report that liver cancer cells show a unique sensitivity toward depletion of fructose-bisphosphate aldolase A (ALDOA) despite other enzymes of the glycolytic pathway being largely dispensable. We find that depletion of ALDOA only has a minor impact on basal glycolytic flux but induces a pronounced build-up of FBP and severe energy exhaustion, indicative of a state of imbalanced glycolysis. Blocking FBP production by ablating the enzyme G6P isomerase (GPI) prevents the induction of imbalanced glycolysis and rescues proliferation in ALDOA-depleted cells. Using a mathematical model of glycolysis, we confirmed that ALDOA inhibition results in imbalanced glycolysis and depletion of inorganic phosphate (Pi). Based on the prediction that lowering glycolytic flux upstream of ALDOA increases the threshold for entering imbalanced glycolysis, we established that reducing glucose availability prevents FBP accumulation and energy exhaustion in ALDOA-depleted cells. Furthermore, downregulation of ALDOA induces pyrimidine deprivation and cell cycle arrest in cancer cells and reduced tumour growth in a mouse model of hepatocellular carcinoma (HCC). These results provide insight into a long-standing question regarding the essentiality of glycolytic enzymes and identify ALDOA as a key metabolic vulnerability of cancer cells engaging in aerobic glycolysis.

## Results

### Common ALDOA dependency across oncogenotypes and conditions

**Fig. 1:**
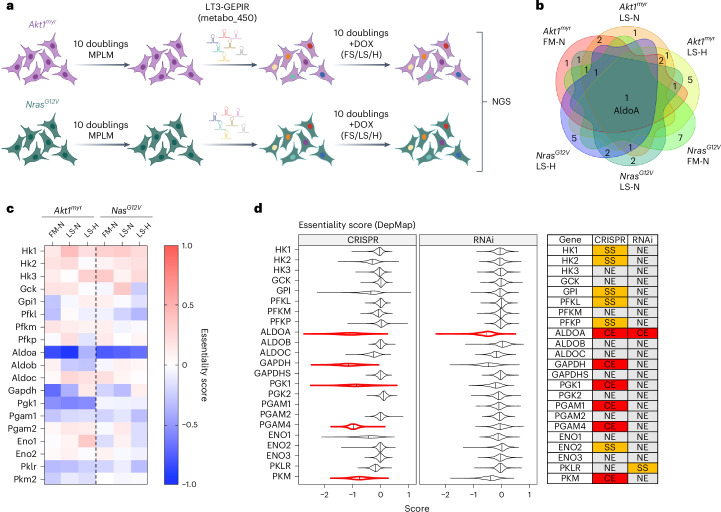
ALDOA represents a unique vulnerability in glycolysis independent of oncogenotype and environmental condition. **a**, Strategy used for the shRNA screen in *Akt1*^*myr*^*;Myc*^*OE*^*;Trp53*^–/–^ or *Nras*^*G12V*^*;Myc*^*OE*^*;Trp53*^–/–^ murine liver cancer cells. Cells were first adapted to MPLM for ten population doublings. After lentiviral transduction, cells were cultured for a further ten population doublings in MPLM containing 10% FCS (FS) or 1% FCS (LS) in an atmosphere containing 20% oxygen (N) or 0.5% oxygen (H). Image created in BioRender (BioRender.com). NGS, next-generation sequencing. **b**, Venn diagram showing the overlap between the ten most essential genes in all cell lines and conditions. Essentiality scores were calculated using MAGeCK. **c**, Heat map of essentiality scores for all glycolytic enzymes represented in the shRNA library in *Akt1*^*myr*^*;Myc*^*OE*^*;Trp53*^–/–^ or *Nras*^*G12V*^*;Myc*^*OE*^*;Trp53*^–/–^ cells across all conditions. **d**, Essentiality scores of all glycolytic enzymes across a large collection of cell lines derived from DepMap. Only *ALDOA* is considered to be ‘common essential’ upon both CRISPR and RNAi-mediated depletion. NE, nonessential; SS, strongly selective.

**Extended Data Fig. 1:**
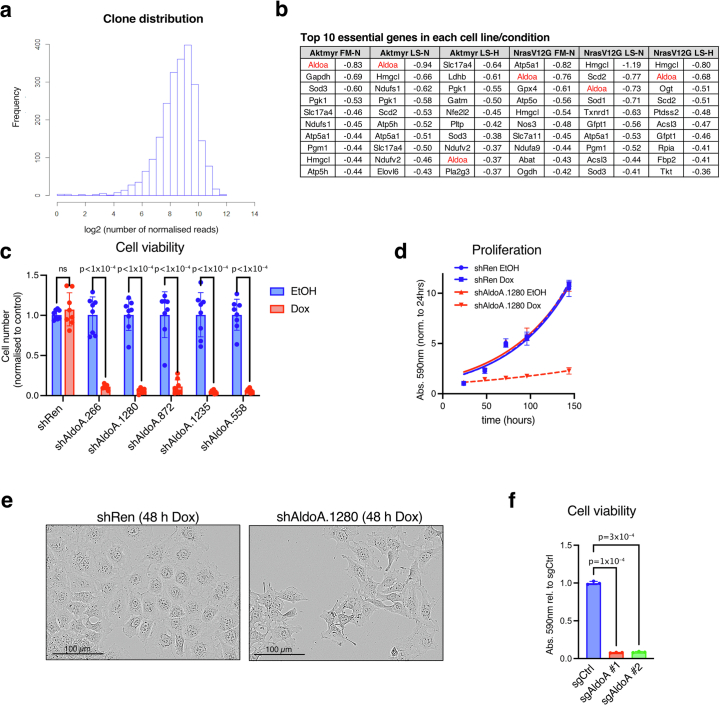
*In vitro* screen identifies ALDOA as essential in HCC cells. **a)** Distribution of individual shRNAs in the shRNA library used for the in vitro screen. **b)** A table showing the 10 top essential genes from the screen for each condition with essentiality scores. Full list is in supplementary table 2. **c)** Viability of *Akt1*^*myr*^*;Myc*^*OE*^*;Trp53*^*–/–*^ cells after 96hrs of AldoA depletion using the five shRNAs against AldoA from the screen. Data are presented as mean values ± SD. Significance was calculated using a two-tailed student’s t-test with FDR correction (n = 8 biologically independent replicates). **d)** Proliferation of control (shRen) or AldoA-depleted (shAldoA.1280) *Akt1*^*myr*^*;Myc*^*OE*^*;Trp53*^*–/–*^ cells in medium containing 1 µg/ml doxycycline (Dox). Data are presented as mean values ± SD (n = 3 biologically independent replicates). **e)** Representative images of control (shRen) or AldoA-depleted (shAldoA.1280) of *Akt1*^*myr*^*;Myc*^*OE*^*;Trp53*^*–/–*^ cells treated with 1 µg/ml doxycycline (Dox) for 48 h. **f)** Cell viability of *Akt1*^*myr*^*;Myc*^*OE*^*;Trp53*^*–/–*^ cells after knockout of Aldoa. Data are presented as mean values ± SD. Significance was calculated using one-way ANOVA with Dunnett’s post-hoc test (n = 3 biologically independent replicates). Source data

The metabolic activity of cancer cells is determined by several factors, including the metabolic characteristics of the parental tissue, the differential activity of oncogenic drivers and the metabolic milieu of the tumour microenvironment^24,25^. To identify metabolic dependencies of cancer cells derived from the same parental tissue but induced by different defined genetic lesions, we employed cell lines derived from murine HCC driven by Akt1/c-Myc/p53^−/−^ (*Akt1*^*myr*^*;Myc*^*OE*^*;Trp53*^–/–^) or by Nras/c-Myc/p53^−/−^ (*Nras*^*G12V*^*;Myc*^*OE*^*;Trp53*^–/–^) generated by transposon-mediated gene transfer in p53-deficient mice^26,27^. The cultures were first adapted to physiological nutrient levels by culturing them in mouse plasma-like medium (MPLM)^28^ for ten population doublings. Next, cells were transduced with a focused short hairpin RNA (shRNA) library targeting approximately 450 genes related to metabolism (average of five hairpins per gene; Extended Data Fig. 1a and Supplementary Table 1). After selection, cells were exposed to different environmental conditions (FM-N, 10% fetal calf serum 21% O_2_; LS-N, 1% fetal calf serum 21% O_2_; and LS-H, 1% fetal calf serum 0.5% O_2_) to simulate tumour-like metabolic stress^29^, cultured for an additional ten population doublings and analysed by next-generation sequencing (Fig. 1a). Comparative evaluation of the ten genes with the highest essentiality score from each condition revealed that only *Aldoa* showed essentiality across both cell lines and under all conditions (Fig. 1b, Extended Data Fig. 1b and Supplementary Table 2). We confirmed that all *Aldoa* shRNA sequences used in the screen were efficient in reducing cell viability (Extended Data Fig. 1c). Furthermore, *Aldoa*-depleted cells showed reduced proliferation and a more spindle-like morphology (Extended Data Fig. 1d,e). In addition, CRISPR/Cas-mediated knockout of *Aldoa* caused a profound reduction in the proliferation of *Myc*^*OE*^*;Akt1*^*myr*^*;Trp53*^*–/–*^ cells (Extended Data Fig. 1f).

The protein encoded by the *Aldoa* gene is a central enzyme in glycolysis. As many cancer cell lines adopt a glycolytic phenotype when cultured in vitro, we interrogated the essentiality scores of all other glycolytic enzymes represented in the library. Notably, none of the genes coding for glycolytic enzymes, apart from *Aldoa*, showed strong essentiality scores in both cell lines and all conditions used (Fig. 1c). Furthermore, interrogation of genetic dependencies across 1,800 cell lines reported in the Cancer Dependency Map (depmap.org) revealed common essentiality (CE) for several glycolytic enzymes, including *ALDOA*, *GAPDH*, *PGK1*, *PGAM4* and *PKM* found by CRISPR-mediated deletion; however, only *ALDOA* was considered to be a CE gene also after gene silencing using RNAi, a targeting strategy unlikely to result in full elimination of the enzyme and only reducing its expression (Fig. 1d). In particular glucose-6-phosphate isomerase (*Gpi*/*GPI)*, for which there is no paralogue in the mouse or human genome, was not identified as essential in our mouse screen nor scored as CE in the DepMap database (Fig. 1c,d). These results indicate that the essentiality of *ALDOA* in cancer cells cannot be explained by an overall requirement of aerobic glycolysis. Instead, *ALDOA* seems to represent a unique metabolic dependency of cancer cells across multiple entities and conditions.

### ALDOA depletion exposes a metabolic bottleneck in glycolysis

**Fig. 2:**
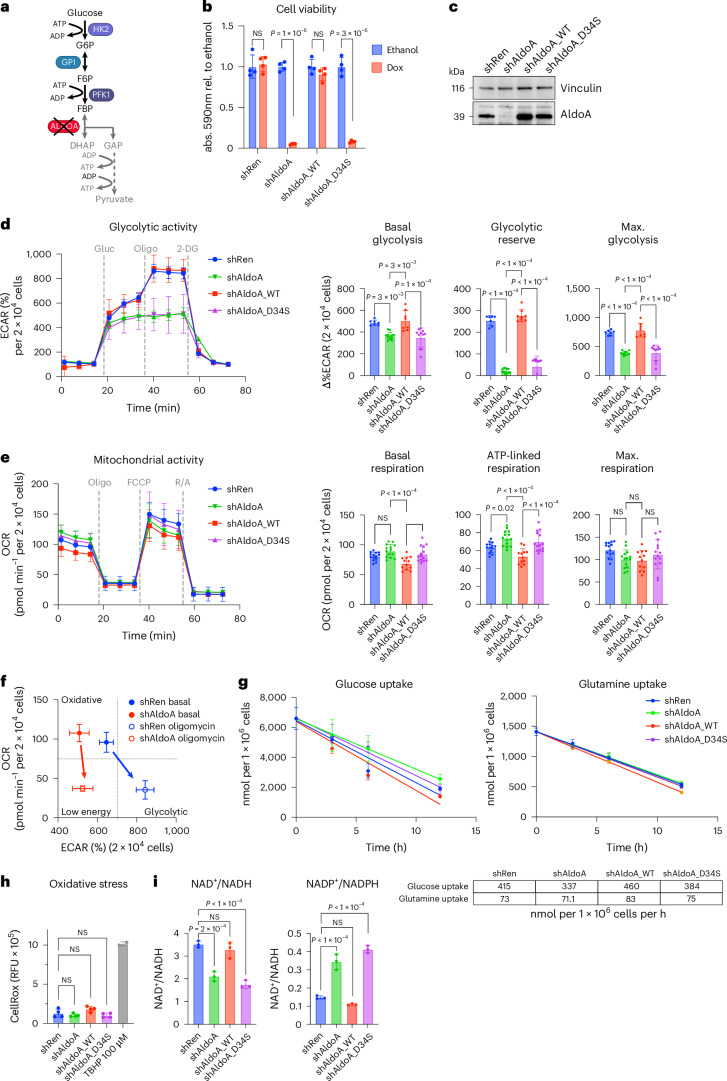
Depletion of ALDOA has minimal effect on basal glycolytic activity. **a**, Diagram of ALDOA in glycolysis. Figure created in BioRender (BioRender.com). **b**, Viability of *Akt1*^*myr*^*;Myc*^*OE*^*;Trp53*^–/–^ cells after ALDOA depletion (shAldoa) or control (shRen) or after silencing and re-expression of WT ALDOA (shAldoa_WT) or a catalytically inactive mutant (shAldoa_D34S). Cells were treated with doxycycline (1 µg ml^−1^) or solvent only (ethanol) for 96 h. Data are presented as mean ± s.d. Significance was calculated using a two-tailed Student’s *t*-test with FDR correction (*n* = 4 biologically independent replicates). **c**, Lysates from cells treated in parallel were analysed for expression of ALDOA. Vinculin is shown as loading control. **d**, Glycolytic activity after 48 h of doxycycline treatment (1 µg ml^−1^). Cells were analysed using the glycolytic stress test (Seahorse). Basal glycolysis, glycolytic reserve and maximal glycolysis are presented as mean ± s.d. Significance was calculated using one-way ANOVA with Dunnett’s post hoc test (shRen: *n* = 7; shAldoa: *n* = 12; shAldoa_WT: *n* = 8; shAldoa_D34S: *n* = 8 parallel wells). The experiment was repeated twice. **e**, Mitochondrial activity in the same cells using the Mito-Stress assay (Seahorse). Basal respiration, ATP-linked respiration and maximal respiration are presented as mean ± s.d. Significance was calculated using one-way ANOVA with Dunnett’s post hoc test (shAldoa_WT: *n* = 12; all others: *n* = 14 parallel wells). The experiment was repeated twice. **f**, Energy map of control (shRen) or ALDOA-depleted cells (shAldo) before and after inhibition of mitochondrial ATP production by oligomycin based on **e**. Data are presented as mean ± s.d. **g**, Glucose and glutamine uptake rates after 48 h of doxycycline treatment (1 µg ml^−1^) and subsequent culture in fresh medium. Data are presented as mean ± s.d. Uptake rates were derived from three biologically independent replicates. **h**, Oxidative stress after 48 h of doxycycline treatment (1 µg ml^−1^) and subsequent culture in fresh medium for 6 h. Data are presented as mean ± s.d. Significance was calculated using one-way ANOVA with Dunnett’s post hoc test (*n* = 3 biologically independent replicates). **i**, NAD^+^/NADH and NADP^+^/NADPH ratios after 48 h of doxycycline treatment (1 µg ml^−1^) and subsequent culture in fresh medium for 6 h. Data are presented as mean ± s.d. Significance was calculated using one-way ANOVA with Dunnett’s post hoc test (*n* = 3 biologically independent replicates). RFU, relative fluorescence units. Source data

**Extended Data Fig. 2:**
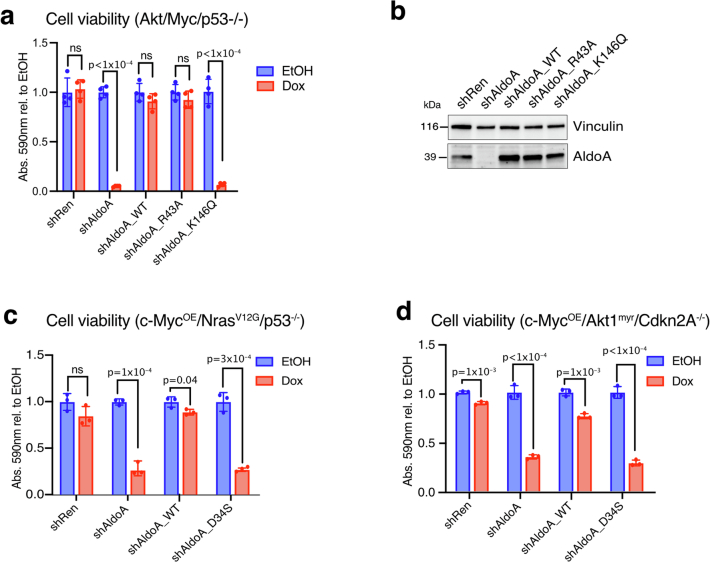
The catalytic function of ALDOA is required for proliferation. **a)** Viability of *Akt1*^*myr*^*;Myc*^*OE*^*;Trp53*^*–/–*^ cells after AldoA depletion (shAldoA) or control (shRen) or after silencing and re-expression of wild type AldoA (shAldoA_WT), a mutant unable to bind to actin (shAldoA_R43A) or a catalytically inactive mutant (shAldoA_K146Q). Inducible silencing and re-expression were achieved by treating cells with doxycycline (1 µg/ml) for 96 h. Data are presented as mean values ± SD. Significance was calculated using a two-tailed student’s t-test with FDR correction (n = 4 biologically independent replicates). **b)** Lysates from the cells shown in a) were analysed for expression of AldoA by Western blotting. Vinculin is shown as loading control. **c)** Viability of *Nras*^*G12V*^*;Myc*^*OE*^*;Trp53*^*–/–*^ cells after AldoA depletion (shAldoA) or control (shRen) or after silencing and re-expression of wild type AldoA (shAldoA_WT) or a catalytically inactive mutant (shAldoA_D34S). Inducible silencing and re-expression were achieved by treating cells with doxycycline (1 µg/ml) for 96 h. Data are presented as mean values ± SD. Significance was calculated using a two-tailed student’s t-test with FDR correction (n = 3 biologically independent replicates). **d)** Viability of *Akt1*^*myr*^*;Myc*^*OE*^*;Cdkn2a*^*ARF*^^*-/-*^ cells after AldoA depletion (shAldoA) or control (shRen) or after silencing and re-expression of wild type AldoA (shAldoA_WT) or a catalytically inactive mutant (shAldoA_D34S). Inducible silencing and re-expression were achieved by treating cells with doxycycline (1 µg/ml) for 96 h. Data are presented as mean values ± SD. Significance was calculated using a two-tailed student’s t-test with FDR correction (n = 3 biologically independent replicates). Source data

The metabolic function of ALDOA is the reversible conversion of FBP into glyceraldehyde 3-phosphate (GAP) and dihydroxyacetone phosphate (DHAP) as part of the glycolytic pathway (Fig. 2a). Many glycolytic enzymes including ALDOA have noncatalytic functions, a phenotype known as ‘moonlighting’^30^. Noncatalytic functions of ALDOA include the regulation of actin dynamics^31^, assembly of lysosomal vATPase^32^ and glucose sensing by AMP activated protein kinase (AMPK)^33^. To investigate whether the essentiality of ALDOA in liver cancer cells involves catalytic or noncatalytic functions, we made use of a shRNA sequence targeting the 3′ nontranslated part of the *Aldoa* mRNA (shAldoa.1280) to combine inducible gene silencing with re-expression of either wild-type (WT) or mutant forms of the enzyme from the same lentiviral vector. Re-expression of WT ALDOA fully restored the viability of *Akt1*^*myr*^*;c-Myc*^*OE*^*;Trp53*^*−/−*^ cells (Fig. 2b,c). Of note, mutation of an arginine residue in the actin-binding domain of ALDOA (R43A), which had previously been shown to be essential for actin binding^34^, had no effect on the ability of ALDOA to support cell viability (Extended Data Fig. 2a,b). In contrast, two different mutants in which the catalytic activity had been deleted (D34S and K146Q) were unable to restore cell viability (Fig. 2b,c and Extended Data Fig. 2a,b). Similar results were also obtained for *Nras*^G12V^;*Myc*^*OE*^*;Trp53*^–/–^ cells and cells derived from *Akt1*^myr^ ;*Myc*^*OE*^ driven, *Cdkn2a*^ARF^-deficient tumours (*Akt1*^myr^;*Myc*^*OE*^*;Cdkn2a*^ARF–/–^) (Extended Data Fig. 2c,d). We therefore concluded that ALDOA essentiality is determined by its catalytic activity rather than its moonlighting functions.

We next explored the effect of ALDOA depletion on metabolic activity. Of note, Seahorse analysis revealed that ALDOA-depleted cells, or cells expressing the catalytically dead mutant D34S, only showed a minor reduction in basal glycolysis, whereas maximal glycolysis and the activation of the glycolytic reserve after blocking mitochondrial ATP production with oligomycin were severely impaired (Fig. 2d). Silencing of *Aldoa* resulted in a small increase in ATP-linked respiration, while basal and maximal respiration were largely unaffected (Fig. 2e). Analysis of the cell energy phenotype before and after oligomycin treatment (Fig. 2f) indicates that, while basal metabolism is not affected, cells depleted of ALDOA are unable to respond to increased energetic demand by increasing their glycolytic flux.

Consistent with the minor effect on basal glycolysis, we also observed that ALDOA depletion had no major effect on glucose or glutamine uptake or oxidative stress (Fig. 2g,h). In contrast, ALDOA-depleted or D43S-expressing cells showed a reduced NAD^+^:NADH ratio and an increased NADP^+^:NADPH ratio (Fig. 2i), potentially as a consequence of metabolic reprogramming in response to ALDOA inactivation.

### Disruption of glycolysis at GPI does not block proliferation

**Fig. 3:**
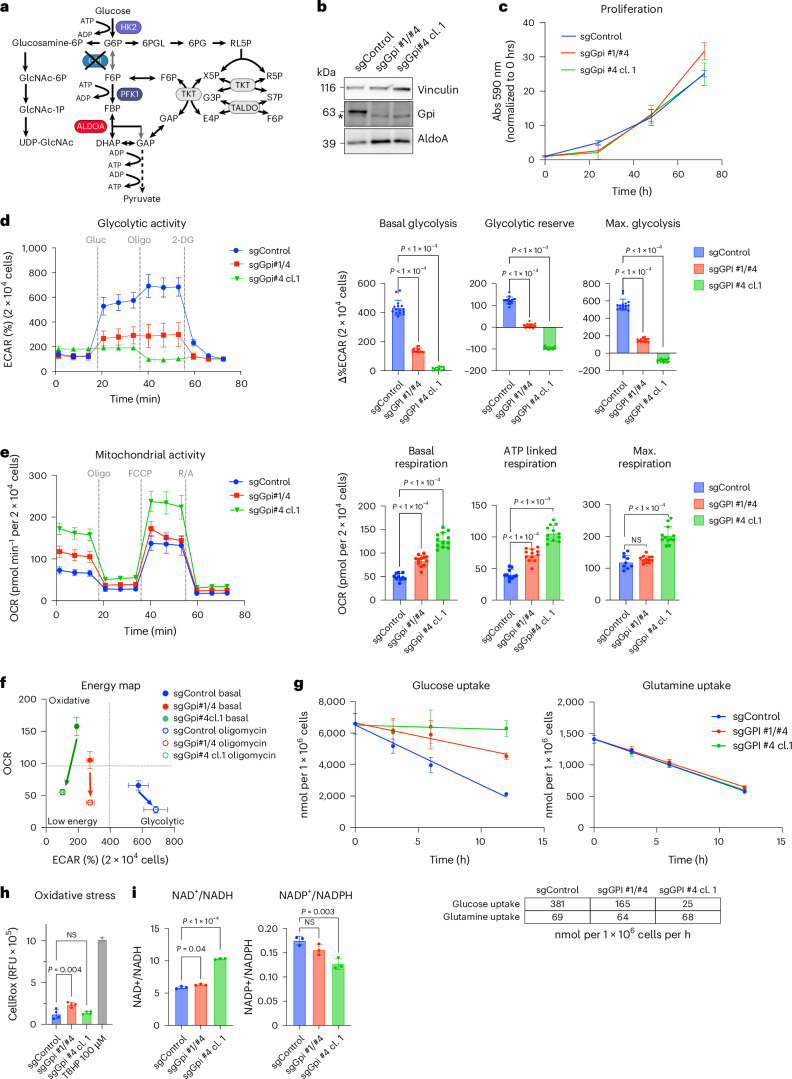
Deletion of *Gpi* does not impair cell proliferation. **a**, Diagram of glycolysis and pentose phosphate shunt indicating disruption of GPI. Figure created in BioRender (BioRender.com). **b**, Detection of GPI in control and *Gpi*-mutant *Akt1*^*myr*^*;Myc*^*OE*^*;Trp53*^–/–^ cells. An unspecific band is indicated by an asterisk. **c**, Proliferation of control (sgControl) and *Gpi* knockout *Akt1*^*myr*^*;Myc*^*OE*^*;Trp53*^–/–^ cells (sgGpi #1/#4 and sgGpi #4 cl.1) (0, 24, 24 h *n* = 6; 72 h *n* = 3 biologically independent experiments). **d**, Glycolytic activity of control (sgControl) and *Gpi* knockout *Akt1*^*myr*^*;Myc*^*OE*^*;Trp53*^–/–^ cells (sgGpi #1/#4 and sgGpi #4 cl.1). Cells were analysed using the glycolytic stress test (Seahorse). Basal glycolysis, glycolytic reserve and maximal glycolysis are presented as mean ± s.d. Significance was calculated using one-way ANOVA with Dunnett’s post hoc test (*n* = 15 parallel wells). The experiment was repeated three times. **e**, Mitochondrial activity was analysed in the same cells as in **d** using the mitochondrial stress test (Seahorse). Basal respiration, ATP-linked respiration and maximal respiration are presented as mean ± s.d. Significance was calculated using one-way ANOVA with Dunnett’s post hoc test (sgControl *n* = 10; sgGpi #1/#4 and sgGpi #4 cl.1 *n* = 12 parallel wells). The experiment was repeated three times. **f**, Energy maps of control or *Gpi* knockout *Akt1*^*myr*^*;Myc*^*OE*^*;Trp53*^–/–^ cells before and after inhibition of mitochondrial ATP production by oligomycin derived from **e**. Data are presented as mean ± s.d. **g**, Glucose and glutamine uptake rates of sgControl and *Gpi* knockout *Akt1*^*myr*^*;Myc*^*OE*^*;Trp53*^–/–^ cells (sgGpi #1/#4 and sgGpi #4 cl.1). Data are presented as mean ± s.d. Uptake rates were derived from three biologically independent replicate cultures. **h**, Oxidative stress of sgControl and *Gpi* knockout *Akt1*^*myr*^*;Myc*^*OE*^*;Trp53*^–/–^ cells (sgGpi #1/#4 and sgGpi #4 cl.1). Data are presented as mean ± s.d. Significance was calculated using one-way ANOVA with Dunnett’s post hoc test (*n* = 3 biologically independent replicates). **i**, NAD^+^/NADH and NADP^+^/NADPH ratio of sgControl and *Gpi* knockout *Akt1*^*myr*^*;Myc*^*OE*^*;Trp53*^–/–^ cells (sgGpi #1/#4 and sgGpi #4 cl.1). Data are presented as mean ± s.d. Significance was calculated using one-way ANOVA with Dunnett’s post hoc test (*n* = 3 biologically independent replicates). Source data

While high glucose utilization is a common metabolic phenotype in cancer, increasing evidence supports the importance of oxidative metabolism for tumour growth^35^. Given that no other glycolytic enzymes showed consistent essentiality in our screens and the DepMap data (Fig. 1c,d), we reasoned that it should be possible to disrupt the glycolytic pathway in cancer cells and still maintain viability. We decided to target the step that converts G6P to F6P, as this preserves the flux into the PPP and hexosamine pathways (Fig. 3a). Furthermore, GPI, the enzyme catalysing this step, has no paralogue in mammalian cells. Indeed, it has previously been shown that depleting GPI suppresses aerobic glycolysis in cancer cells without affecting cell proliferation in vitro and only a minimal effect on tumour growth^36^.

**Extended Data Fig. 3:**
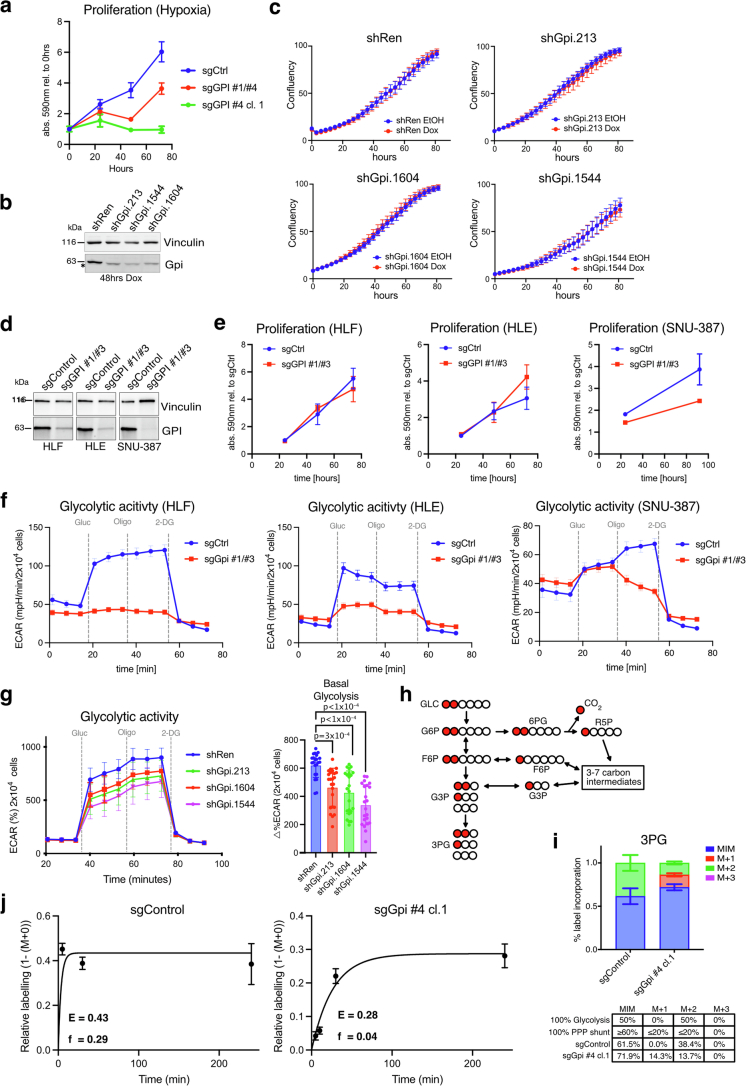
Deletion of GPI leads to metabolic reprogramming. **a)** Proliferation of control (sgControl) and GPI knockout *Akt1*^*myr*^*;Myc*^*OE*^*;Trp53*^*–/–*^ cells (sgGPI #1/#4 and sgGPI #4 cl.1) cultured under hypoxia (0.5% O_2_). Data are displayed as mean ± SD (0 h n = 4, all others n = 8 biologically independent replicates). **b)** Western blot probing for GPI and Vinculin after 48hrs doxycycline (1ug/mL) treatment of shRen, shGPI.213, shGPI.1604 and shGPI.1544 expressing *Akt1*^*myr*^*;Myc*^*OE*^*;Trp53*^*-/-*^ cells. **c)** Proliferation of shRen, shGPI.213, shGPI.1604 and shGPI.1544 expressing *Akt1*^*myr*^*;Myc*^*OE*^*;Trp53*^*–/–*^ cells treated with doxycycline (1 µg/mL) or EtOH (solvent). Confluency was detected using live cell imaging of parallel wells. Data are presented as mean values ± SD (shRen and shGPI.155 EtOH n = 11, all others n = 12 parallel wells). **d)** Deletion of Gpi in human liver cancer cells (HLF, HLE and SNU-387) using CRISPR/Cas9. **e)** Proliferation of human liver cancer cells after depletion of GPI (sgGPI #1/#3) or control (sgCtrl). Cell mass was detected by crystal violet staining of independently seeded wells. Data are presented as mean values ± SD (HLE: n = 9; SNU387 sgGPI #1/#3 92 h n = 2, all others n = 3; HLF n = 8). **f)** Glycolytic activity of control (sgControl) and GPI knockout cells (sgGPI #1/#3) Cells were analysed using the glycolytic stress test (Seahorse). Data are presented as mean values ± SD (n = 15 parallel wells). **g)** Glycolytic activity of shRen, shGPI.213, shGPI.1604 and shGPI.1544 expressing *Akt1*^*myr*^*;Myc*^*OE*^*;Trp53*^*–/–*^ cells. Cells were analysed using the glycolytic stress test (Seahorse). Data are presented as mean values ± SD. Significance was calculated using one-way ANOVA with Dunnett’s post-hoc test (shRen and shGPI.1604 n = 21, all others n = 23 parallel wells). **h)** Diagram indicating the flux of ^13^C-labelled carbon atoms from 1,2-^13^C-Glucose into glycolysis and the pentose phosphate pathway (PPP). **i)** Isotopologue distribution at steady state for 3-phophoglycerate (3PG) in control cells (sgControl) and Gpi deleted cells (sgGpi #1/#4 or sgGPI #4 cl. 1). Table shows the estimated isotopologue distribution for 3PG assuming 100% flux through glycolysis or 100% flux through PPP^69^ in addition to the observed experimental results. Data are presented as mean values ± SD (n = 2 biologically independent replicates). **j)** Non-linear fit of relative enrichment of ^13^C labelled 3PG after 5, 10, 30 and 240 min of culture in 1,2-^13^C-Glucose containing medium, (E) is maximum enrichment at isotopic steady state and (f) is calculated flux. Data are displayed as mean ± SD (n = 2 biologically independent replicates). Source data

Genetic deletion of *Gpi* was achieved using CRISPR/Cas9 technology either with two combined guide RNA (gRNA) sequences (sgGpi#1/#4) in a pooled cell population or by selecting a knockout clone generated by a single gRNA (sgGpi#4 cl.1) (Fig. 3b). In concordance with previous findings^36^, deletion of *Gpi* had no effect on cell proliferation of murine liver cancer cells cultured in physiological medium conditions in normoxia (Fig. 3c); however, these cells were unable to adapt to hypoxic conditions (Extended Data Fig. 3a). To exclude effects caused by clonal adaptation, we also employed gene silencing using doxycycline-inducible shRNA expression. This confirmed that acute silencing of *Gpi* did not impair cell viability (Extended Data Fig. 3b,c). Similarly, *GPI* knockout did not impair proliferation in three human liver cancer cell lines (HLE, HLF and SNU-387) (Extended Data Fig. 3d,e). Seahorse analysis revelated that stable *Gpi* knockout or acute silencing caused a severe reduction in basal glycolysis and completely blocked the mobilization of the glycolytic reserve in both murine and human liver cancer cells (Fig. 3d and Extended Data Fig. 3f,g).

The impact of *Gpi* deletion on glycolytic flux was also investigated by [1,2]^13^C-glucose tracing into 3-phosphoglycerate (3PG) (Extended Data Fig. 3h). Stable isotope distribution at steady state (240 min) showed a higher proportion of M + 1 labelled 3PG, confirming rewiring of glycolytic flux via the oxidative and nonoxidative arms of the PPP (Extended Data Fig. 3i). However, while the glycolytic flux was evidently rewired, the overall rate of label incorporation into glycolytic metabolites was strongly reduced in *Gpi* knockout cells (28.6% versus 3.8% ^13^C enrichment per min), confirming inhibition of the pathway (Extended Data Fig. 3j). In parallel to the reduction in glycolytic flux, *Gpi* knockout cells showed a marked upregulation of basal, ATP-linked and maximal respiration (Fig. 3e), indicating that cells undergo a shift from a glycolytic to an oxidative phenotype. This was in contrast to ALDOA depletion, where no increase in OXPHOS was observed (Fig. 2e). In addition, *Gpi* knockout cells were no longer able to induce glycolysis in response to oligomycin treatment (Fig. 3f). We also observed a strong reduction in glucose uptake in *Gpi*-deleted cells. This was not accompanied by a compensatory upregulation of glutamine uptake (Fig. 3g), indicating that the cells generate ATP much more efficiently by complete oxidation of glucose. Alternatively, *Gpi*-deleted cells could compensate by upregulating fatty acid oxidation. Despite the increased respiration observed in *Gpi* knockout cells, there was no increase in oxidative stress (Fig. 3h). Furthermore, we observed an increase in the NAD^+^:NADH ratio which is in agreement with increased mitochondrial metabolism. In contrast, the NADP^+^:NADPH ratio is decreased in *Gpi* knockout cells, which could be a consequence of increased flux through the oxidative PPP (Fig. 3i). Together, these results suggest that disruption of glycolysis at the level of GPI has no major impact on cell viability and proliferation by allowing cancer cells to undergo a metabolic switch to ATP production via increased mitochondrial respiration.

### Blocking ALDOA catalytic activity induces severe energy stress

**Fig. 4:**
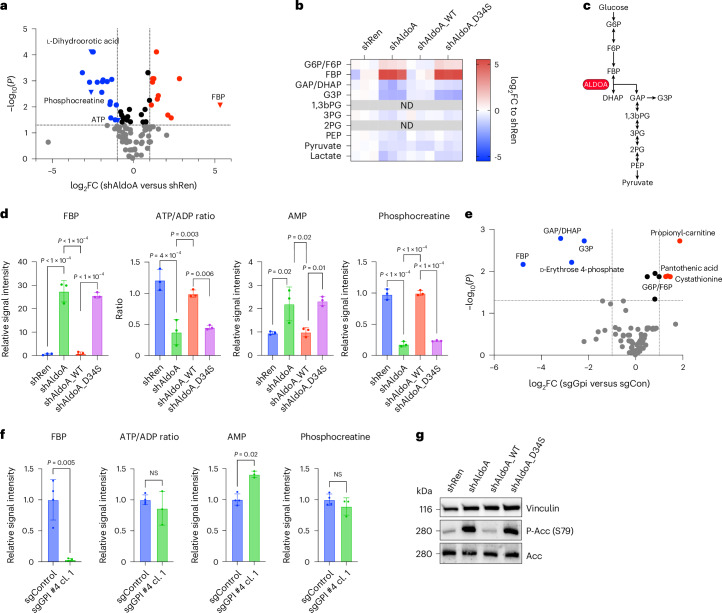
Depletion of ALDOA exposes a metabolic bottleneck in glycolysis leading to severe energy exhaustion. **a**, Volcano plot comparing intracellular metabolites of control (shRen) or ALDOA-depleted (shAldo.1280) *Akt1*^*myr*^*;Myc*^*OE*^*;Trp53*^–/–^ cells after 48 h of doxycycline treatment. Cells were collected 6 h after addition of fresh MPLM medium. Significance was calculated using a two-tailed Student’s *t*-test with FDR correction (*n* = 3 biologically independent replicates). **b**, Heatmap showing the log_2_ fold change (FC) for several glycolytic intermediates in ALDOA-depleted (shAldoa.1280) or control (shRen) cells and in cells re-expressing WT (shAldoa_WT) or catalytically inactive ALDOA (shAlddoa_D34S) (*n* = 3 biologically independent replicates). ND, not determined. **c**, Diagram of glycolytic intermediates detected in **b**. Figure created in BioRender (BioRender.com). **d**, Intracellular metabolite levels for FBP, phosphocreatine and AMP as well as the ATP:ADP ratio in ALDOA-depleted (shAldoa) or control (shRen) cells and in cells re-expressing WT (shAldoa_WT) or catalytically inactive ALDOA (shAlddoa_D34S). Data are presented as mean ± s.d. Significance was calculated using one-way ANOVA with Dunnett’s post hoc test (*n* = 3 biologically independent replicates). **e**, Volcano plot comparing intracellular metabolites of control (sgControl) or *Gpi* knockout (sgGpi #4 cl.1) *Akt1*^*myr*^*;Myc*^*OE*^*;Trp53*^–/–^ cells. Significance was calculated using a two-tailed Student’s *t*-test with FDR correction (*n* = 3 biologically independent replicates). **f**, Intracellular metabolite levels for FBP, phosphocreatine and AMP as well as the ATP:ADP ratio in control (sgControl) or *Gpi* knockout (sgGpi #4 cl.1) cells. Data are presented as mean ± s.d. Significance was calculated using one-way ANOVA with Dunnett’s post hoc test (*n* = 3 biologically independent replicates). **g**, Activation of AMPK in control (shRen) and ALDOA (shAldoa)-depleted cells and after expression of WT (shAldoa_WT) or catalytically inactive ALDOA (shAldoa_D34S). AMPK activation was determined by monitoring serine 79 phosphorylation in ACC. Source data

**Extended Data Fig. 4:**
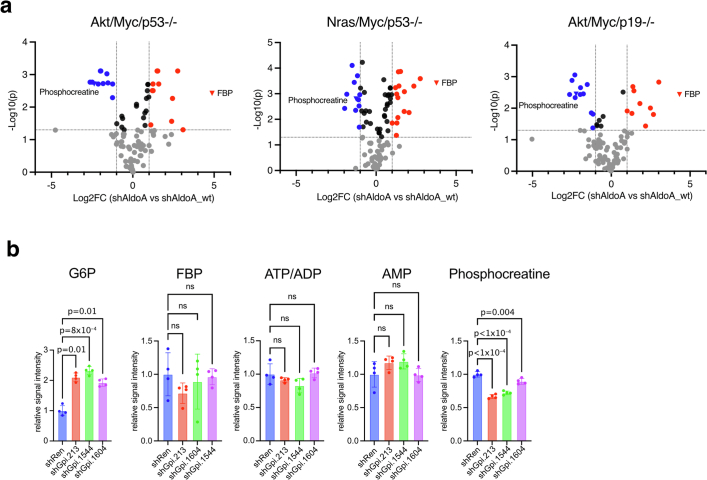
Metabolic reprogramming after ALDOA and GPI depletion. **a)** Volcano plots comparing intracellular metabolites of AldoA depleted cells (shAldoA.1280) and AldoA re-expression (shAldoA_WT) in *Akt1*^*myr*^*;Myc*^*OE*^*;Trp53*^*–/–*^, *Nras*^*G12V*^*;Myc*^*OE*^*;Trp53*^*–/–*^ and *Akt1*^*myr*^*;Myc*^*OE*^*;Cdkn2a*^*ARF-/-*^ cells after 48 h of doxycycline treatment. Cells were harvested 6 h after addition of fresh MPLM media. Names of selected metabolites are shown. Significance was calculated using a two-tailed students t-test with FDR correction (n = 3 biologically independent replicates). **b)** Intracellular metabolite levels for G6P, FBP, phosphocreatine and AMP as well as the ATP/ADP ratio in shRen, shGPI.213, shGPI.1604 and shGPI.1544 expressing *Akt1*^*myr*^*;Myc*^*OE*^*;Trp53*^*–/–*^ cells treated with doxycycline (1 µg/mL) for 48hrs. Data are presented as mean values ± SD. Significance was calculated using one-way ANOVA with Dunnett’s post-hoc test (n = 4 biologically independent replicates). Source data

Having observed the stark differences in cellular response to targeting glycolysis at the level of either GPI or ALDOA, we next determined changes in cellular metabolite levels using liquid chromatography (LC) linked to mass spectrometry (MS). For this, *Akt1*^*myr*^*;c-Myc*^*OE*^*;Trp53*^*−/−*^ cells were first depleted of ALDOA by inducing RNAi expression for 48 h and then treated with fresh medium for 6 h before analysis. LC–MS analysis revealed that the most strongly upregulated metabolite was FBP, the substrate of ALDOA, with levels being >30-fold higher compared with control cells (Fig. 4a). FBP was also the strongest upregulated metabolite in *Nras*^*G12V*^*;c-Myc*^*OE*^*;Trp53*^*−/−*^ and *Akt1*^*myr*^*;c-Myc*^*OE*^*;Cdkn2a*^*ARF*^^*–/–*^cells following ALDOA depletion (Extended Data Fig. 4a). Re-expression of WT ALDOA, but not the catalytic mutant D34S, prevented the strong accumulation of FBP (Fig. 4b). Closer inspection of metabolic intermediates of glycolysis showed that while GAP/DHAP and glycerol 3-phosphate (G3P) were downregulated, metabolites of lower glycolysis were only mildly affected (Fig. 4b,c). In addition, ALDOA depletion resulted in marked reduction of ATP, a significant decrease in the ATP:ADP ratio and increased levels of AMP (Fig. 4a,d), indicating profound exhaustion of cellular energy. Moreover, phosphocreatine, a readily available source of high-energy phosphoryl-groups used for the rapid regeneration of ATP from ADP, was strongly reduced (Fig. 4a,d). The dramatic loss of phosphocreatine after depletion of ALDOA was restored by re-expression of the WT enzyme but not the catalytically inactive mutant (Fig. 4d).

In contrast, cells depleted of GPI and treated in the same manner showed a significant downregulation of several glycolytic intermediates, most notably FBP (Fig. 4e). However, despite the complete disruption of glycolytic flux, *Gpi* knockout cells showed no significant change in ATP/ADP ratio or phosphocreatine levels, with only a small increase in AMP levels (Fig. 4f). Comparable results were also obtained after acute silencing of *Gpi* using inducible shRNA expression, with the notable exception that levels of FBP were not lowered, potentially due to a compensatory upregulation of G6P. Moreover, phosphocreatine levels were slightly reduced, indicating incomplete metabolic adaptation after acute GPI depletion (Extended Data Fig. 4b).

As disruption of the catalytic activity of ALDOA caused a severe reduction in cellular energy charge, we next investigated whether this also resulted in the activation of AMPK, a master regulator of metabolic homeostasis in response to low energy^37^. Indeed, FBP and ALDOA have been shown to be involved in glucose sensing by AMPK independent of AMP/ADP^33^. We observed that depletion of ALDOA induced serine 79 phosphorylation of acetyl-CoA carboxylase (ACC), indicative of AMPK activation. Furthermore, re-expression of the catalysis-defective D34S mutant, which retains the activity to bind FBP and prevent AMPK activation in glucose-starved HEK293 cells and MEFs^33^, did not avert AMPK activation in murine liver cancer cells exposed to glucose-replete medium (Fig. 4g). This finding supports the conclusion that blocking the catalytic activity of ALDOA in cancer cells under conditions where glucose is readily available leads to a severe state of energy deprivation that overrides the glucose-sensing mechanism of AMPK.

### Depletion of ALDOA induces imbalanced glycolysis

**Fig. 5:**
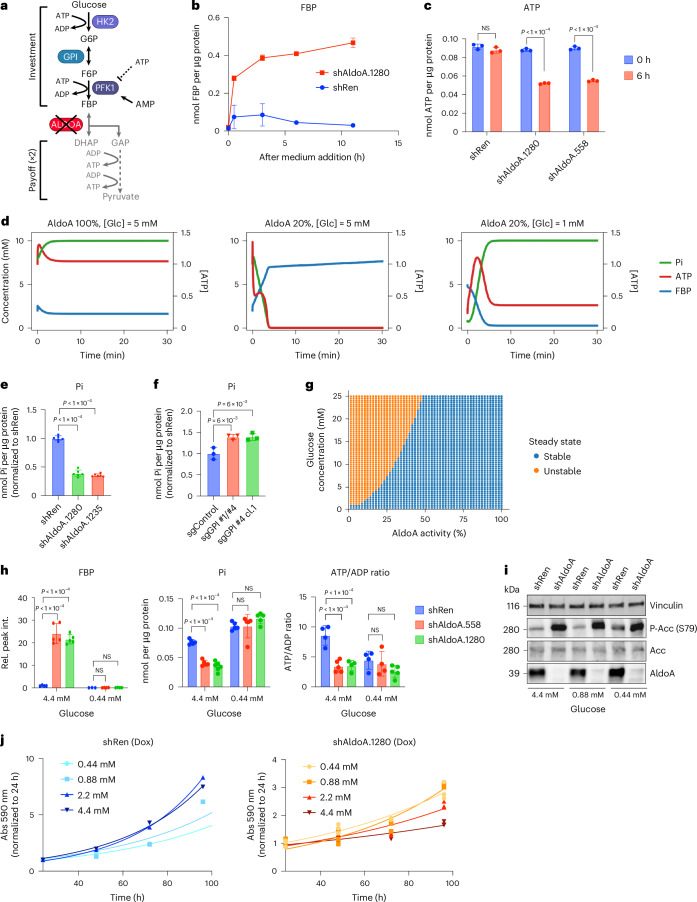
Deletion of ALDOA leads to imbalanced glycolysis. **a**, Diagram depicting the autocatalytic structure of glycolysis and allosteric regulation of PFK1. Figure ceated in BioRender (BioRender.com). **b**, FBP levels in control (shRen) or ALDOA-depleted (shAldoA.1280) *Akt1*^*myr*^*;Myc*^*OE*^*;Trp53*^–/–^ cells after 48 h doxycycline treatment (1 µg ml^−1^) followed by addition of fresh medium including doxycycline for indicated times. Data are presented as mean ± s.d. (*n* = 3 biologically independent replicates). **c**, ATP levels in control (shRen) or ALDOA-depleted (shAldoa.1280, shAldoa.558) cells after 48 h of doxycycline treatment (1 µg ml^−1^) followed by addition of fresh medium for 6 h. Data are presented as mean ± s.d. Significance was calculated using a two-tailed Student’s *t*-test with FDR correction (*n* = 3 biologically independent replicates). **d**, Dynamic mathematical model of glycolysis simulating levels of FBP, ATP and Pi in controls (ALDOA 100% glucose 5 mM, left), ALDOA-depleted (ALDOA 20% glucose 5 mM, middle) or ALDOA-depleted cells after glucose deprivation (ALDOA 20% glucose 1 mM, right). **e**, Pi in control (shRen) or ALDOA-depleted (shAldoa.1280, shAldoa.1235) cells treated as in **a**. Data are presented as mean ± s.d. Significance was calculated using one-way ANOVA with Dunnett’s post hoc test (*n* = 6 independent biological replicates). **f**, Pi in control (sgControl) or *Gpi* knockout (sgGpi #1/#4, sgGpi #4 cl.1) cells after addition of fresh medium for 6 h. Data are presented as mean ± s.d. Significance was calculated using one-way ANOVA with Dunnett’s post hoc test (*n* = 3 biologically independent replicates). **g**, Bifurcation analysis. Diagram shows the parameter regions corresponding to stable or unstable states as a function of *V*_max_, *ALD* and [GLC_e_]. Maximal ALDOA activity is expressed as a percentage of reference value. **h**, Levels of FBP and Pi as well as the ATP:ADP ratio in control (shRen) and ALDOA-depleted (shAldoa.558 and shAldoa.1280) cells after incubation in fresh MPLM medium containing 4.4 mM or 0.44 mM glucose for 6 h. Data are presented as mean ± s.d. Significance was calculated using one-way ANOVA with Dunnett’s post hoc test (biologically independent replicates: *n* = 5 shAldoa.558 4.4 mM and shAldoa.1280 0.44 mM, *n* = 4 others). **i**, Activation of AMPK determined by phospho-ACC (S79) in control (shRen) and ALDOA-depleted (shAldoa.1280) cells after culture in medium containing indicated glucose concentrations for 6 h. Vinculin is shown as loading control. **j**, Proliferation of control (shRen) or ALDOA-depleted (shAldoa.1280) cells in medium containing indicated glucose concentrations. Data are presented as mean ± s.d. (*n* = 3 biologically independent replicates). Source data

Glycolysis is controlled at the level of PFK1 through allosteric activation and inhibition by AMP and ATP, respectively, matching glycolytic flux with cellular energy demand (Fig. 5a). As strong FBP accumulation and severe energy stress in ALDOA-depleted cells was observed after 6 h of exposure to fresh medium containing all nutrients, including glucose, we reasoned that it could be the consequence of high glucose availability. We therefore determined FBP levels in control and ALDOA-depleted cells after 48 h of silencing in the same medium and over a time course of medium re-addition. In line with previous findings^33^, control cells respond to renewed glucose availability by a moderate and transient increase in FBP (Fig. 5b). Remarkably, ALDOA-depleted cells showed the same initial low levels of FBP as control cells but responded to medium addition with strong and sustained FBP accumulation (Fig. 5b). Furthermore, ATP levels were initially similar in control and ALDOA-depleted cells, but were strongly reduced in ALDOA-depleted cells at 6 h after addition of fresh medium (Fig. 5c).

**Extended Data Fig. 5:**
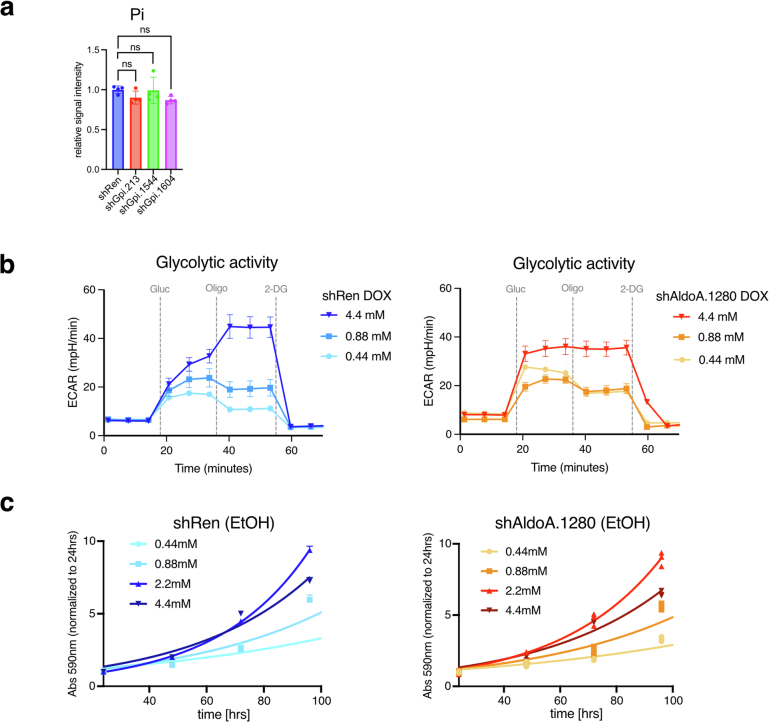
The effects of ALDOA depletion are partially ameliorated by culture in low glucose. **a)** Intracellular levels of inorganic phosphate (Pi) in shRen, shGPI.213, shGPI.1604 and shGPI.1544 expressing *Akt1*^*myr*^*;Myc*^*OE*^*;Trp53*^*–/–*^ cells treated with doxycycline (1 µg/mL) for 48hrs. Data are presented as mean values ± SD. Significance was calculated using one-way ANOVA with Dunnett's post-hoc test (n = 4 biologically independent replicates). **b)** Glycolytic activity of control (shRen) and AldoA knockdown cells (shAldoA) under the indicated glucose concentrations. Cells were analysis using the glycolytic stress test (Seahorse). Data are presented as mean values ± SD (n = 6 parallel wells). **c)** Proliferation of control (shRen) or AldoA (shAldoA.1280) cells in medium containing ethanol (solvent) and indicated glucose concentrations. Data are presented as mean values ± SD (n = 3 biologically independent replicates). Source data

The initially counterintuitive finding that provision of fresh medium containing glucose to ALDOA-depleted cells could, in a matter of hours, lead to a strong reduction in ATP levels coinciding with a prominent build-up of FBP is reminiscent of imbalanced glycolysis, a phenotype caused by the loss of HK regulation by glycolysis and previously observed in yeast^16^. To further explore the relationship between glucose availability, glycolytic flux and ALDOA catalytic activity we made use of a coarse-grained mathematical model of glycolysis based on ordinary differential equations (Supplementary Information). The model describes flux through the glycolytic pathway, as well as levels of ATP, FBP and inorganic phosphate (Pi), and is capable of reproducing the observed results for a broad range of parameters. Specifically, at 100% ALDOA activity, the model gives rise to a viable stable steady state with low FBP levels (Fig. 5d, left graph), consistent with what was observed in WT cells (Fig. 5b); however, when ALDOA activity is reduced to 20%, the model gives rise to an imbalanced glycolytic state that is characterized by accumulation of FBP to high levels and severe depletion of ATP and Pi. As reported previously^16^, and also observed here (Fig. 5d, middle graph), the marked drop in Pi is caused by trapping high-energy phosphate groups in metabolites of upper glycolysis, most notably FBP. Consistent with the model, experimental ALDOA depletion in *Akt1*^*myr*^*;c-Myc*^*OE*^*;p53*^−/−^ cells resulted in a marked reduction in Pi (Fig. 5e). In contrast, knockout of *Gpi* caused an increase in Pi levels (Fig. 5f), inversely matching the low FBP levels observed in these cells (Fig. 4f). Similarly, acute silencing of *Gpi*, which did not lower FBP, did not affect Pi levels (Extended Data Fig. 5a).

Based on these findings, we considered potential mechanisms to rebalance glycolysis in ALDOA-depleted cells. The coarse-grained model indicates that reducing glycolytic flux by lowering glucose availability restores the balance between upper and lower glycolysis and prevents FBP accumulation (Fig. 5g). For example, 20% of ALDOA activity is sufficient to support balanced glycolysis when glucose concentrations are lowered to 1 mM (Fig. 5d, right graph). We therefore investigated whether reduced glucose provision could prevent the induction of imbalanced glycolysis. We therefore exposed control and ALDOA-depleted cells to fresh medium containing either at physiological (4.4 mM) or reduced (0.44 mM) concentrations of glucose for 6 h. This revealed that lowering the glucose availability prevented the accumulation of FBP and fully restored intracellular Pi levels in ALDOA-depleted cells (Fig. 5h); however, cells were unable to recover energy levels under glucose starvation, as the ATP:ADP ratio was reduced also in control cells (Fig. 5h) and AMPK was activated under all conditions (Fig. 5i). Moreover, glucose deprivation did not restore the induction of the glycolytic reserve in ALDOA-depleted cells (Extended Data Fig. 5b). We next explored the effect of different glucose concentrations on proliferation in control and ALDOA-depleted cells. While solvent-only-treated cells or cells expressing a nontargeting shRNA (shRen) responded to glucose starvation by reducing growth, proliferation of ALDOA-depleted cells was slightly enhanced under low-glucose concentrations (Fig. 5j and Extended Data Fig. 5c). These results indicate that, while glucose starvation prevents the induction of imbalanced glycolysis, it is not sufficient to support the high metabolic demand of cancer cells during proliferation.

### Combined block of GPI and ALDOA prevents imbalanced glycolysis

**Fig. 6:**
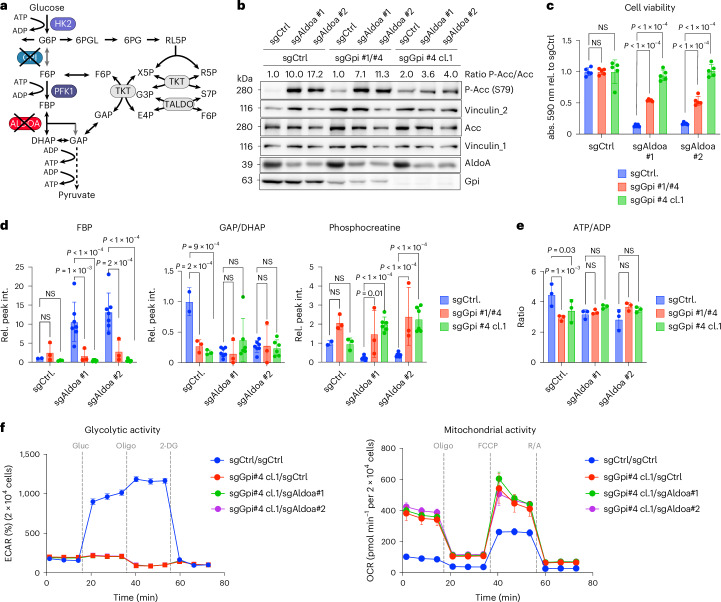
Combined depletion of GPI and ALDOA rescues imbalanced glycolysis and restores proliferation. **a**, Diagram indicating combined deletion of GPI and ALDOA. Created in https://BioRender.com. RL5P, ribulose-5-phosphate. **b**, Immunoblot confirming genetic deletion of *Aldoa* in control and *Gpi* knockout *Akt1*^*myr*^*;Myc*^*OE*^*;Trp53*^–/–^ cells using two nonoverlapping sgRNAs (sgAldoa #1 and sgAldoa #2). Activation of AMPK was determined by analysing serine 79 phosphorylation on ACC. Vinculin is shown as loading control. **c**, Cell viability of *Akt1*^*myr*^*;Myc*^*OE*^*;Trp53*^–/–^ cells after genetic deletion of *Aldoa* or after combined deletion of *Aldoa* and *Gpi*. Data are presented as mean ± s.d. Significance was calculated using one-way ANOVA with Dunnett’s post hoc test (*n* = 5 independent biological replicates). **d**, Intracellular metabolite levels for FBP, GAP/DHAP and phosphocreatine in *Akt1*^*myr*^*;Myc*^*OE*^*;Trp53*^*–/–*^ cells after deletion of *Aldoa* or after combined deletion of *Aldoa* and *Gpi*. Data are presented as mean ± s.d. Significance was calculated using one-way ANOVA with Dunnett’s post hoc test (number of independent biological replicates: *n* = 3 for sgCtrl *n* = 6 for sgAldoa#1 and sgAldoa#2). **e**, ATP:ADP ratio in *Akt1*^*myr*^*;Myc*^*OE*^*;Trp53*^–/–^ cells after deletion of *Aldoa* or after combined deletion of *Aldoa* and *Gpi*. Data are presented as mean ± s.d. Significance was calculated using one-way ANOVA with Dunnett’s post hoc test (*n* = 3 independent biological replicates). **f**, Glycolytic and mitochondrial activity of control (sgControl) and *Gpi* knockout *Akt1*^*myr*^*;Myc*^*OE*^*;Trp53*^–/–^ cells (sgGpi #4 cl.1) or cells with combined deletion of *Gpi* and *Aldoa*. Cells were analysed using the glycolytic or mitochondrial stress test (Seahorse). Data are presented as mean ± s.d. (sgGpi#4 cl.1/sgCtrl *n* = 12, others *n* = 11 parallel wells). Source data

**Extended Data Fig. 6:**
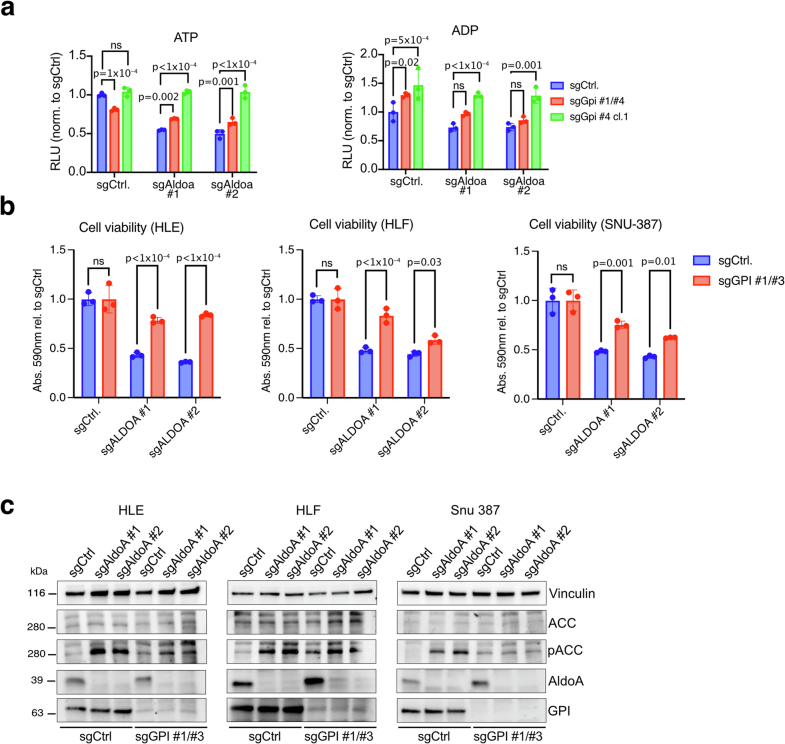
Deletion of GPI rescues the impact of ALDOA deletion in human HCC cells. **a)** ATP and ADP levels in *Akt1*^*myr*^*;Myc*^*OE*^*;Trp53*^*–/–*^ cells after deletion of AldoA or after combined deletion of *Aldoa* and *Gpi*. Data are presented as mean values ± SD. Significance was calculated using one-way ANOVA with Dunnett's post-hoc test (n = 3 biologically independent replicates). **b)** Cell viability of human liver cancer cells (HLE, HLF and SNU-387) after deletion of *GPI* (sgGPI #1/#3) either alone or on combination with *ALDOA*- (sgALDOA #1, sgALDOA #2). Data are presented as mean values ± SD. Significance was calculated using a two-tailed student’s t-test with FDR correction (n = 3 biologically independent replicates). **c)** Immunoblot confirming the deletion of *GPI* (sgGPI #1/#3) either alone or on combination with *ALDOA* (sgALDOA #1, sgALDOA #2) in human liver cancer cells (HLE, HLF and SNU-387). Activation of AMPK was determined by analysing S79 phosphorylation on ACC. Vinculin is shown as loading control. Source data

To confirm that inhibition of cell viability following ALDOA depletion is mechanistically linked to the induction of imbalanced glycolysis, we hypothesized that genetic deletion of *Gpi* should prevent the trapping of high-energy phosphate groups in FBP (Fig. 6a). We therefore combined CRISPR-mediated deletion of *Aldoa* and *Gpi* in murine cancer cells using several combinations of gRNAs (Fig. 6b). As predicted by our model, deletion of *Gpi* fully restored cell viability in *Aldoa* knockout cells (Fig. 6c). This was accompanied by a complete block in FBP accumulation and a strong reduction in GAP/DHAP levels (Fig. 6d). We also established levels of ATP and ADP in single and double-knockout cells and found that combined deletion of *Gpi* and *Aldoa* did not fully restore the ATP:ADP ratio to that of control cells, most likely as *Gpi* deletion already reduced this ratio despite having no effect on cell proliferation. We also observed that *Aldoa* deletion reduced the amounts of both ATP and ADP, which was restored by co-deletion of *Gpi* (Extended Data Fig. 6a). Nevertheless, deletion of *Gpi* strongly increased phosphocreatine levels in *Aldoa*-deleted cells, indicative of restored cellular energy levels (Fig. 6d,e). Furthermore, Seahorse analysis showed that cells depleted of GPI and ALDOA showed impaired glycolytic activity but a strong upregulation of mitochondrial activity (Fig. 6f). This confirms that blocking glycolytic flux upstream of PFK prevents the engagement of the regulatory feedback loop that leads to the establishment of imbalanced glycolysis and severe energy stress. It also allows upregulation of mitochondrial metabolism to compensate for glycolytic impairment. Notably, knockout of *Gpi* also prevented the induction of ACC phosphorylation in response to *Aldoa* deletion, further confirming the restoration of cellular energy levels (Fig. 6b). We also extended this analysis to human liver cancer cell lines (HLE, HLF and SNU-387). Crucially, deletion of *GPI* rescued the effect of *ALDOA* deletion by significantly increasing cell number and, similar to the murine system, attenuating AMPK activation (Extended Data Figs. 6b,c).

Together, these data demonstrate that blocking glycolysis at the step catalysed by ALDOA results in the trapping of high-energy phosphate in upper glycolysis. Under these conditions, cells enter a state of imbalanced glycolysis that results in profound energy stress. Imbalanced glycolysis is aggravated by allosteric activation of PFK, inducing a vicious cycle that further increases the synthesis of FBP, thereby depleting intracellular phosphate stores and blocking proliferation. Preventing the accumulation of large amounts of FBP by blocking GPI averts imbalanced glycolysis and restores viability.

### Rewiring of metabolism in ALDOA-depleted cells

To obtain deeper insight into the metabolic consequences of imbalanced glycolysis we applied stable-isotope tracing. We initially conducted time-resolved experiments by treating control and ALDOA-depleted cells with 4.4 mM of fresh glucose for 30 or 180 min to induce imbalanced glycolysis. After this time, the medium was replaced with medium containing ^13^C_6_-glucose and cells were incubated for a further 5, 10 or 15 min. This allowed us to determine flux for the reaction from glucose to G6P as well as changes in the abundance of the M + 6 isotopologues of G6P, F6P and FBP. In addition, we monitored the abundance of the M + 3 isotopologue of G3P, produced from glycolytic intermediates downstream of ALDOA, and the M + 5 isotopologue of R5P, a product of the oxidative PPP. It should be noted that it was not possible to determine flux for all reactions of upper glycolysis, as these are not in metabolic steady state under these conditions.

**Fig. 7:**
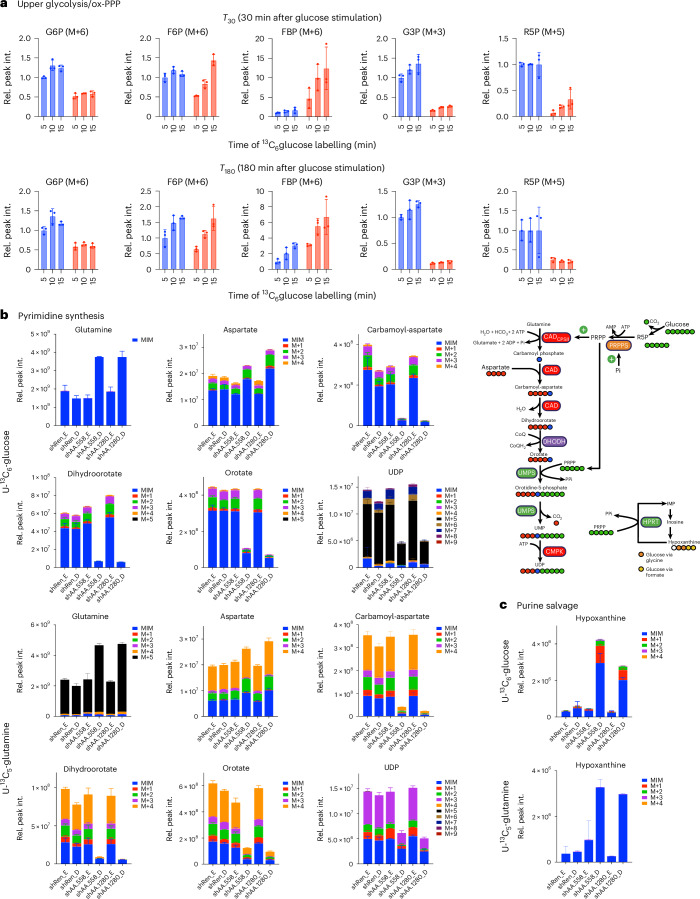
Depletion of ALDOA retains flux in upper glycolysis but impairs oxidative PPP and pyrimidine synthesis. **a**, shRen and shAldoa *Akt1*^*myr*^*;Myc*^*OE*^*;Trp53*^–/–^ cells were treated with 1 µg ml^−1^ doxycycline for 48 h before fresh medium containing 4.4 mM glucose and doxycycline was added for 30 min (*T*_30_) or 180 min (*T*_180_). Subsequently, cells were cultured in medium containing 4.4 mM U-^13^C_6_-glucose for 5, 10 or 15 min. Metabolites were extracted and analysed by LC–MS. Graphs show relative peak intensity of the isotopologue reflecting full labelling normalized to shRen (5 min) for the indicated metabolites. Data are presented as mean ± s.d. (*n* = 3 independent biological replicates). **b***, Akt1*^*myr*^*;Myc*^*OE*^*;Trp53*^–/–^ cells expressing shRNA sequences targeting *Aldoa* (shAldoA.558 and shAldoA.1280) or nontargeting controls (shRen) were treated with 1 µg m^−1^l doxycycline (D) or solvent (ethanol, E) for 48 h before fresh medium containing 4.4 mM glucose including doxycycline or solvent was added for 1 h. Subsequently, cells were exposed to medium containing either 4.4 mM U-^13^C_6_-glucose or 1 mM U-^13^C_5_-glutamine for 10 h. Metabolites were extracted and analysed by LC–MS. Graphs show pool size and labelling of metabolic substrates and intermediates of the pyrimidine biosynthesis pathway. The diagram shows the carbons derived from aspartate, bicarbonate and R5P to uridine diphosphate (UDP). Data are presented as mean ± s.d. (*n* = 3 independent biological replicates). **c**, Pool size and labelling of hypoxanthine from the same samples as in **b**. Diagram shows synthesis of inosine monophosphate (IMP) from hypoxanthine via the purine salvage pathway. Data are presented as mean ± s.d. (*n* = 3 independent biological replicates). Source data

**Extended Data Fig. 7:**
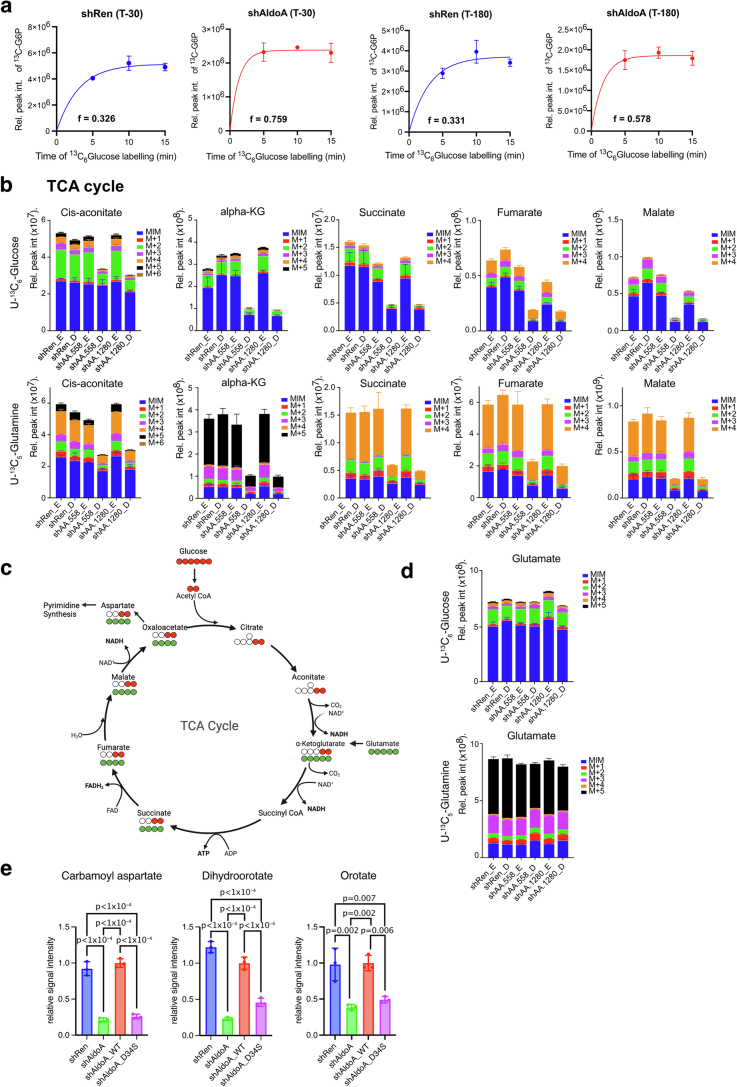
Depletion of ALDOA has minimal effect on glycolytic flux but impairs the TCA cycle. **a)** Non-linear fit of absolute enrichment of ^13^C labelled G6P after 5, 10 or 15 min of culture in U-^13^C-Glucose containing medium, (f) is calculated flux. Data are displayed as mean ± SD (n = 3 biologically independent replicates). **b)** *Akt1*^*myr*^*;Myc*^*OE*^*;Trp53*^*–/–*^ cells expressing shRNA sequences targeting AldoA (shAldoA.558 and shAldoA.1280) or non-targeting controls (shRen) were treated with 1 µg/ml doxycycline (D) or solvent (ethanol, E) for 48 hrs before fresh medium containing 4.4 mM glucose including doxycycline or solvent was added for 1 h. Subsequently, cells were exposed to medium containing either 4.4 mM U-^13^C_6_-glucose or 1 mM U-^13^C_5_-glutamine for 10 h. Metabolites were extracted and analysed by LC-MS. Graphs show pool size and labelling of TCA cycle intermediates. Data are displayed as mean ± SD (shAA588_E n = 2, all others n = 3 biologically independent replicates). **c)** Diagram of the TCA cycle showing carbon labelling from glucose and glutamine. **d)** Pool size and labelling of glutamate in the same samples as in (b). Data are displayed as mean ± SD (shAA588_E n = 2, all others n = 3 biologically independent replicates). **e)** Intracellular metabolite levels for carbamoyl aspartate, dihydroorotate and orotate in AldoA depleted (shAldoA) or control (shRen) cells and in cells re-expressing wild type (shAldoA_WT) or catalytically inactive AldoA (shAlddoA_D34S). Data are presented as mean values ± SD. Significance was calculated using one-way ANOVA with Dunnett’s post-hoc test (n = 3 biologically independent replicates). Source data

The results of this analysis showed that the flux from glucose into G6P was higher in ALDOA-depleted cells compared with controls, albeit with reduced abundance of the M + 6 isotopologue (Extended Data Fig. 7a and Fig. 7a). Furthermore, we observed a strong accumulation of the M + 6 isotopologue of FBP in ALDOA-depleted cells, particularly at the 30-min time point, confirming that ALDOA-depleted cells continue to synthesize this metabolite (Fig. 7a). In contrast, the production of metabolites downstream of ALDOA was severely impaired, as we observed a strong reduction in the M + 3 isotopologue of G3P. Interestingly, this analysis also revealed a substantial reduction in oxidative PPP, as the M + 5 isotopologue of R5P was strongly reduced. Together, these results confirm our model in which the continued production of FBP in ALDOA-depleted cells leads to the trapping of high-energy phosphate and severe energy stress.

We also conducted experiments using labelling at isotopic steady state by culturing control and ALDOA-depleted cells with ^13^C_6_-glucose or ^13^C_5_-glutamine. After treating cells with doxycycline for 48 h to achieve ALDOA silencing, cells were induced to enter imbalanced glycolysis by addition of fresh medium containing 4.4 mM glucose for 1 h before addition of the labelled nutrient for 10 h. Isotopologue analysis showed that incorporation of carbons derived from glucose or glutamine into the TCA cycle was reduced in ALDOA-depleted cells and the total pool size of all measured TCA cycle intermediates was strongly diminished (Extended Data Fig. 7b,c). In contrast, levels and isotopologue distribution of glutamate remained mostly unchanged (Extended Data Fig. 7d). This indicates that the flux of metabolites into the TCA cycle is reduced but intermediates are still being drained into biosynthetic processes.

Nucleotide synthesis is among the cellular metabolic processes that require large amounts of energy and are regulated by the availability of phosphate^38^. In particular, the first steps of pyrimidine synthesis respond to changes in ATP and Pi due to allosteric regulation of phosphoribosyl pyrophosphate synthetase and the multifunctional protein CAD. Given the strong antiproliferative effect of ALDOA depletion, we next investigated the synthesis of pyrimidine nucleotides, which require TCA cycle-derived carbons in the form of aspartate and nitrogen derived from the glutamine amide moiety. Of note, total metabolite levels of aspartate and glutamine were increased in ALDOA-depleted cells (Fig. 7b), indicative of a reduced consumption of these metabolites by the first and second step of pyrimidine biosynthesis, catalysed by the CAD enzyme. Subsequent metabolites of the pyrimidine biosynthesis pathway, including dihydroorotate and orotate, were drastically depleted. Moreover, re-expression of ALDOA WT, but not the catalysis-defective D34S mutant, restored levels of these metabolites (Extended Data Fig. 7e). CAD activity is regulated by the availability of phosphoribosyl pyrophosphate (PRPP), the synthesis of which requires large amounts of both ATP and R5P and is controlled by intracellular Pi^39^. Consistently, we observed a strong accumulation of hypoxanthine, an intermediate of the purine salvage pathway (Fig. 7c), indicating that the recycling of purine bases by hypoxanthine phosphoribosyl transferase, which requires PRPP as substrate, is blocked in ALDOA-depleted cells. Together, these results support the conclusion that imbalanced glycolysis leads to an inhibition of nucleotide biosynthesis due to a lack of PRPP and ATP.

### ALDOA depletion induces S-phase arrest and blocks tumour growth

**Fig. 8:**
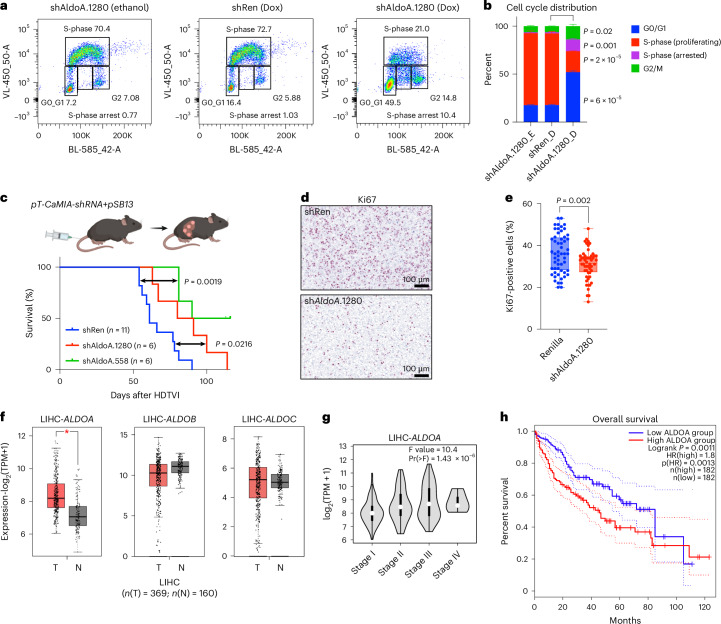
Targeting of ALDOA blocks cell cycle progression and increases survival in an autochthonous mouse model of liver cancer. **a**, Cell cycle distribution of control (shRen) or ALDOA-depleted (shAldoa.1280) *Akt1*^*myr*^*;Myc*^*OE*^*;Trp53*^–/–^ cells after 48 h of doxycycline treatment (1 µg ml^−1^) followed by addition of fresh MPLM medium for 6 h. Cells were incubated with 10 µM of BrdU for 30 min before collection. **b**, Quantification of cell cycle distribution in control and ALDOA-depleted cells. Data are presented as mean ± s.d. Significance was calculated using multiple two-tailed unpaired *t*-tests with FDR correction (Benjamini–Krieger–Yekutieli, *n* = 3 biologically independent experiments). **c**, Survival analysis of C57BL/6 mice injected with *pT-CaMIA-shRen* or *pT-CaMIA-shAldoa* transposons together with Sleeping Beauty transposase (pSB13) by hydrodynamic tail vein injection (HDTVI). Groups were compared by two-tailed chi-squared test (Gahan–Beslow–Wilcoxon). **d**, Representative histological sections from control (shRen) and AldoA-depleted murine liver tumours stained for the proliferation marker Ki67. **e**, Quantification of histological sections using QPath. *pT-CaMIA-shRen* (*n* = 3 mice) and *pT-CaMIA-shAldoa* (*n* = 5 mice). Significance was calculated using a two-tailed Student’s *t*-test. Six tumour nodules representing individual transformation events were analysed per mouse. Data are displayed as box plot (box, 25th to 75th percentile; whiskers, min to max). **f**, Comparison of RNA expression for *ALDOA*, *ALDOB* and *ALDOC* in human LIHC samples from The Cancer Genome Atlas (TCGA) (*n* = 369) and corresponding normal liver from the Genotype Tissue Expression (GTEx) project (*n* = 160). Analysis was performed using GEPIA2 with a *P* value cutoff of 0.01 (in all cases indicated by a single asterisk the *P* values were below this cutoff) using a one-way ANOVA and the results are presented as a boxplot. T, tumour; N, normal. **g**, Expression of *ALDOA* in LIHC according to the disease stage. Data were derived from TCGA and analysed using GEPIA2 (ref. ^65^). **h**, Survival analysis (Kaplan–Meier plots) for patients with liver cancer with high and low *ALDOA* expression using GEPIA2. Source data

**Extended Data Fig. 8:**
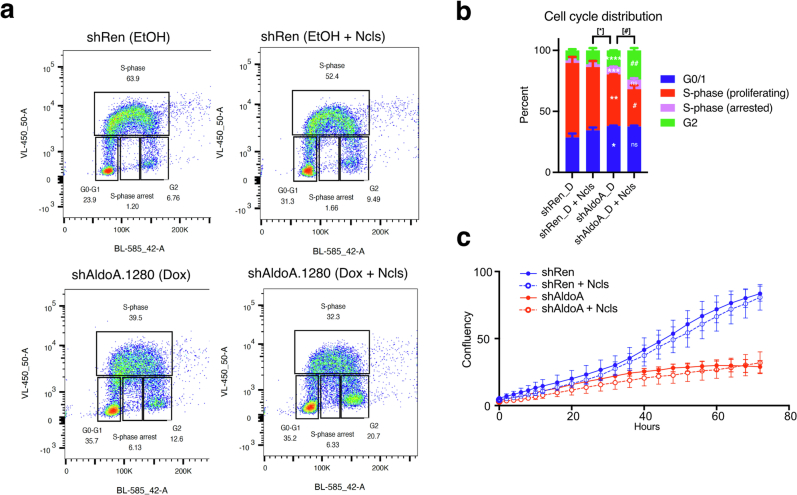
ALDOA depletion cannot be rescued by nucleoside addition. **a)** Cell cycle distribution of control (shRen) or Aldo depleted (shAldoA.1280) *Akt1*^*myr*^*;Myc*^*OE*^*;Trp53*^*–/–*^ cells after 48 h of doxycycline treatment (1 µg/ml) followed by addition of fresh medium with or without nucleosides added for 6 h. Cells were incubated with 10 µM of BrdU for 0.5 h before harvest. **b)** Quantification of cell cycle distribution in control and AldoA depleted cells with or without addition of nucleosides. Data are presented as mean values ± SD. Significance was calculated using multiple two-tailed unpaired t-tests with Benjamini-Krieger-Yekutieli FDR correction (n = 3 biologically independent replicates, *p = 0.0075, **p = 0.0027, ***p = 0.00057, ****p = 0.0008, #p = 0.003, ##p = 0.0006). **c**) Proliferation of shRen and shAldoA *Akt1*^*myr*^*;Myc*^*OE*^*;Trp53*^*–/–*^ cells treated with doxycycline (1 µg/mL) and cultured with or without nucleosides. Data are displayed as mean ± SD (shRen n = 3, all others n = 4 parallel wells). Source data

Having observed a strong effect of imbalanced glycolysis on nucleotide biosynthesis, we next determined the effect of ALDOA depletion on cell cycle distribution (Fig. 8a,b). This revealed a marked reduction in the proportion of cells undergoing active DNA synthesis (from 70% to 20%) accompanied by an increase in the proportion of cells in G0/G1 and G2. Of note, cells also showed evidence of S-phase arrest, a phenotype associated with nucleotide depletion^40^; however, addition of nucleosides did not prevent S-phase arrest and did not restore proliferation in ALDOA-depleted cells (Extended Data Fig. 8a–c).

We next explored the consequences of ALDOA depletion in a well-established genetically engineered liver cancer mouse model^27^. We used hydrodynamic tail vein injection of transposons encoding c-*Myc* and *Akt1*^*myr*^ together with shRNAs targeting *Aldoa* or nontargeting control (*pT-CaMIA-shRNA*). Both *Aldoa* shRNAs (shAldoa.1280 and shAldoa.558) strongly reduced tumour development and prolonged survival of tumour-bearing animals in comparison to a nontargeting control shRNA (shRen) (Fig. 8c), indicating that ALDOA is essential for liver tumour development. This is particularly remarkable, as this model was previously shown to display resistance toward the multikinase inhibitor sorafenib^27^, a first-line therapy for HCC. Moreover, tumours arising after ALDOA depletion showed a marked reduction in proliferation, indicated by lower Ki67 positivity (Fig. 8d,e). Finally, analysis of public data indicates that *ALDOA* is highly expressed in The Cancer Genome Atlas-liver hepatocellular carcinoma (TCGA-LIHC) samples (Fig. 8f,g) and a strong predictor of poor survival (Fig. 8h). This is in agreement with earlier reports indicating an isoform switch from ALDOB to ALDOA in liver cancer^41,42^. Together, these results suggest that inducing imbalanced glycolysis by targeting ALDOA could be a therapeutic strategy for cancers that engage in aerobic glycolysis.

## Discussion

In this work we found that the glycolytic enzyme aldolase A is essential for proliferation in glycolytic cancer cells even though the glycolytic pathway itself is dispensable. Indeed, ALDOA was the only glycolytic enzyme that showed CE both in our RNAi screen in murine HCC cell lines with different oncogenotypes cultured under diverse environmental conditions and across the multiple cancer cell lines represented in the Cancer Dependency Map. Disrupting the catalytic activity of ALDOA was accompanied by strong accumulation of FBP and severe energy stress despite only causing a minor reduction in basal glycolysis. In contrast, blocking glycolysis at the level of Gpi was fully tolerated by murine and human cancer cells, rendering them also insensitive to ALDOA depletion. Moreover, we found that depletion of ALDOA caused reduced cancer cell proliferation and extended survival in a mouse model of HCC.

The strong dependency of cancer cells on the catalytic activity of ALDOA is caused by the autocatalytic nature of glycolysis and the crucial position of ALDOA in the sequence of reactions of the glycolytic pathway, controlling the transit of metabolites from the investment phase of upper glycolysis into the energy-producing reactions (payoff phase) of lower glycolysis. Inhibition of ALDOA catalytic activity in highly glycolytic cancer cells thus leads to the induction of an imbalanced glycolytic state that effectively transforms glycolysis from an ATP-producing into an ATP-consuming pathway. Mechanistically, when glucose enters glycolysis in a cell depleted of ALDOA, the glycolytic intermediate FBP cannot be further metabolized by lower glycolysis and rapidly accumulates. There are three major reasons why imbalanced glycolysis is an extremely problematic situation for the cell. (1) The conversion of F6P to FBP catalysed by PFK is essentially irreversible under physiological conditions. In addition, the only enzymes that utilize FBP as substrate are aldolase and FBPase, the latter being exclusively expressed in gluconeogenic tissues (such as the healthy liver) and frequently downregulated in cancer^2,43,44^. (2) FBP, the end product of the investment phase of glycolysis, is an energy-rich molecule, as it contains both of the two phosphates invested into glycolysis by hexokinase and PFK1, respectively. Thus, strong accumulation of FBP, as seen in ALDOA-depleted cells, leads to profound trapping of high-energy phosphate groups and subsequent energy depletion. (3) Energy depletion caused by imbalanced glycolysis activates PFK, thus causing a vicious cycle in which reduced ATP levels lead to increased ATP consumption by upper glycolysis and a further build-up of FBP. As a consequence, cells can only escape this state of imbalanced glycolysis by limiting the influx into upper glycolysis, thus reducing the production of FBP, for example under conditions of reduced glucose availability or in response to Gpi deletion.

Previous studies describing imbalanced glycolysis as a result of loss of HK inhibition in yeast have reported that the resulting uncontrolled activation of upper glycolysis leads to a strong reduction in intracellular levels of Pi and ATP due to FBP synthesis acting as a trap for high-energy phosphate groups. Furthermore, the reduced availability of inorganic phosphate creates a blockage at the level of glyceraldehyde-3-phosphate dehydrogenase (GAPDH), which further compounds the problem^16,45^. A similar relationship between FBP accumulation and reduced ATP and Pi levels was also observed in our coarse-grained kinetic model of glycolysis. Experimentally, we confirmed the glucose-dependent increase in FBP as well as a reduction in ATP and Pi as predicted by the model. Furthermore, ALDOA-depleted cells continued to consume glucose and convert it into FBP, as demonstrated by glucose-uptake experiments and stable-isotope tracing; however, unlike in both our model and the model describing imbalanced glycolysis caused by removal of HK inhibition^16^, we did not observe a complete depletion of ATP and Pi levels in ALDOA-depleted cells. The likely cause for this discrepancy is that the models do not account for compartmentalization of these metabolites between the cytosol and mitochondria (and possibly other compartments) or the contribution of respiration toward ATP production. Indeed, we found that although ALDOA-depleted cells had strongly reduced ATP levels, the overall activity of respiration was largely unaffected, even displaying a small but significant increase in ATP-linked respiration. This is in stark contrast to the effect of Gpi deletion, which caused a pronounced increase in both basal and ATP-linked respiration, in line with previous observations in melanoma and colon adenocarcinoma cells^36^. A similar shift from glycolysis to oxidative phosphorylation has also been observed in cells in which glycolysis was inhibited by culture in acidic medium^46,47^, which inhibits glycolysis at the level of PFK1 (refs. ^47,48^). This phenotype is reminiscent of an inversion of the Crabtree effect, a principle describing the suppression of oxidative phosphorylation by high glycolytic activity^49^. Inhibition of upper glycolysis at the level of Gpi could relieve the inhibition of oxidative phosphorylation by the Crabtree effect, thus allowing cells to compensate for the loss of glycolytic ATP production. Notably, targeting glycolysis at the level of ALDOA does not allow this compensatory activation of oxidative phosphorylation. This could potentially be mediated by high FBP levels, as studies in yeast have indicated that FBP inhibits components of the electron transport chain and has already been discussed as a mediator of the Crabtree effect^50,51^; however, other metabolites, including inorganic phosphate^52^ or intermediates of lower glycolysis (GAP, 3-phosphoglycerate and phosphoenolpyruvate)^53^, have also been proposed to induce electron transport chain inhibition.

In addition to its catalytic function in glycolysis, ALDOA has important noncanonical functions. Aldolases have been shown to bind to the actin cytoskeleton^34,54,55^ and this interaction was found to be inhibited by the presence of FBP^56^. However, we observed that expression of the R43A mutant of ALDOA, which renders the enzyme catalytically active but unable to bind to actin^54^, was fully capable of supporting cell proliferation in cells depleted of endogenous ALDOA. This indicates that the ability of ALDOA to bind actin is nonessential for proliferation in our system. A second noncanonical function of ALDOA involves the regulation of AMPK and mTORC1 (refs. ^33,57,58^). It was shown that aldolase bound to its substrate FBP inhibits AMPK activity. Of note, the D34S mutation in ALDOA mimics FBP-bound ALDOA, as it is catalytically inactive but can nevertheless mediate signalling to prevent AMPK activation. We found that expression of ALDOA D34S did not prevent AMPK activation upon in cells depleted of endogenous ALDOA, indicating that AMPK can still be activated by energy stress even though it receives the signal that glycolysis is active. In contrast, we found that Gpi deletion led to basal AMPK activation even though the energetic state of the cells was only mildly affected, suggesting that AMPK might also sense low glycolytic activity via the absence of FBP, as previously reported^33^, as FBP levels were very low in GPI-deleted cells. These two modes of AMPK activation (via sensing of low glycolysis or via detection of energy stress) could lead to different metabolic outputs and GPI-deleted cells could provide an experimental system in which this question could be addressed.

Using stable-isotope tracing, we confirmed that flux in upper glycolysis is maintained in ALDOA-depleted cells and that the cells continue to synthesize FBP; however, we also found that ALDOA depletion leads to a reduction in the oxidative PPP, which could be caused by allosteric activation of PFK. In addition, we observed a strong reduction in the synthesis of pyrimidine nucleotides, most likely caused by the combination of reduced levels of ATP, loss of inorganic phosphate and depletion of R5P due to imbalanced glycolysis. This resulted in acute cell cycle inhibition, including S-phase arrest. Pyrimidine biosynthesis is governed by the CAD enzyme, which is allosterically activated by PRPP^39^. PRPP itself is synthesized from ATP and R5P by PRPP synthetase whose activity is highly dependent on Pi, thus providing a link between intracellular phosphate availability and proliferation^59^. Moreover, salvage of purine nucleotides was also blocked at the level of PRPP incorporation. We therefore conclude that the antiproliferative effect of imbalanced glycolysis in cancer cells could be due to inhibition of nucleotide biosynthesis.

Our finding that ALDOA is a common essential enzyme in HCC cell lines across different oncogenotypes and during exposure to diverse environmental conditions highlights the high susceptibility of cancer cells to imbalanced glycolysis. This could also be a mechanistic explanation for the unique essentiality of ALDOA observed across hundreds of human cancer cell lines in the Cancer Dependency Map, a phenotype not shared by other glycolytic enzymes. Aerobic glycolysis is a metabolic hallmark in many cancer entities, including HCC, and could be exploited for cancer therapy^60^. Preclinical studies have revealed promising effects of targeting glycolysis by deletion of HK2 in lung and breast cancer^19^. However, it is clear that the ability of cancer cells to shift from glycolysis to oxidative phosphorylation for ATP production presents a major challenge for therapeutic strategies blocking glycolysis and may require combinatorial treatments^61^. Targeting ALDOA presents an opportunity to overcome the inherent metabolic plasticity of cancer cells, as it not only removes the ability of cells to utilize glycolysis as an energy-generating pathway but converts glycolysis into a trap for high-energy phosphate. Inducing imbalanced glycolysis by targeting ALDOA could thus be highly efficient in eliminating cancer cells.

## Methods

### Cell culture

Murine liver cancer cells were cultured in murine plasma-like medium (MPLM), an ‘in-house’-made medium based on the composition of mouse plasma as described in ref. ^28^ with 10% dialysed fetal bovine serum, 100 U ml^−1^ penicillin and 100 μg ml^−1^ streptomycin. The formulation of MPLM is provided in Supplementary Table 4. To assess the impact of nucleoside addition on proliferation and cell cycle, the medium was supplemented with 1× Embryomax (ES-008-D Merck). Human liver cancer cell lines were kindly provided by J.P.F. Angeli and cultured in DMEM with 10% fetal bovine serum, 4 mM l-glutamine, 100 U ml^−1^ penicillin and 100 μg ml^−1^ streptomycin (all Sigma). All cell lines were cultured at 37 °C in a humidified incubator at 5% CO_2_.

### Library preparation and genetic screening

The shRNA library consists of 2,258 shRNAs targeting 457 metabolic genes. The mirE-based shRNAs were designed by J. Zuber (Research Institute of Molecular Pathology, Vienna Biocenter) ordered as 97-mer DNA oligonucleotides from IDT and cloned into *pLT3GEPIR* using XhoI or EcoRI restriction sites^62^. Sequence representation was confirmed by next-generation sequencing. shRNA sequences are provided in Supplementary Table 1. *Akt1*^*myr*^*;c-Myc*^*OE*^*;Trp53*^*−/−*^ and c-Myc^OE^/*Nras*^*G12V*^*;c-Myc*^*OE*^*;Trp53*^*−/−*^ cells were transduced by lentiviral infection (multiplicity of infection of 0.3 and clonal redundancy of 1,000) and selected with puromycin for 3 days, at which stage a sample of genomic DNA was taken (*T*_0_). Cells were then cultured in different conditions for ten population doublings while maintaining shRNA representation and genomic DNA was prepared (*T*_1_). Experiments were performed in biologically independent triplicate. Normalized read counts generated from next-generation sequencing were analysed to identify essential genes using MAGeCK^63^. Essentiality scores are provided in Supplementary Table 2.

### Genetic modification using shRNA and CRISPR

For inducible gene silencing, shRNA sequences targeting murine *Aldoa* were cloned into *pLT3GEPIR* (Addgene, #111177). Viral particles were produced in HEK293T cells and stable populations were selected using puromycin. Sequences are provided in Supplementary Table 1. For CRISPR/Cas9-mediated deletion of murine and human *GPI* and human *ALDOA*, sgRNA sequences were designed using CHOPCHOP (https://chopchop.cbu.uib.no) or VBC score (https://www.vbc-score.org) and cloned into *lentiCRISPRv2* (Addgene, #52961) or *LentiGuide-TagRFP-2A-BSD* (Addgene, #167930). Murine *Aldoa* sgRNA sequences originate from ref. ^64^ (Addgene, #1000000052). Cells were infected and selected with the respective agent and either used as pools or single clones, as indicated. Silencing and knockout efficiency was confirmed using western blotting. Sequences are provided in Supplementary Table 3.

### Analysis of cell viability and proliferation using crystal violet or live imaging

Cells were seeded in 24- or 96-well plates and treated as indicated. After incubation, cells were washed with PBS and fixed for 10 min in 3.7% paraformaldehyde. Cells were washed and stained with 0.1% crystal violet solution (Sigma) for 1 h. Plates were rinsed in water, dried and extracted using 10% acetic acid. Absorbance was measured at 550 nm. To monitor cell proliferation, cells were seeded on 96-well plates and confluency was monitored over time using live-cell imaging (Incucyte, Sartorius).

### Western blot analysis

Cells were lysed in RIPA buffer (150 mM NaCl, 50 mM Tris, pH 8.0, 1% (v/v) NP-40, 0.5% (w/v) sodium deoxycholate and 0.1% (w/v) SDS) with protease and phosphatase inhibitors for 30 min and cleared by centrifugation. Proteins were quantified using BCA (Thermo Scientific). Proteins were separated on SDS–PAGE and blotted onto PVDF membrane (Immobilon), treated with blocking solution (5% BSA) and incubated with primary and secondary antibodies in 5% BSA. Signals were detected on a ChemiDoc (Bio-Rad). The antibodies used were anti-ALDOA (11217-1-AP, Proteintech), anti-GPI (15171-1-AP, Proteintech or CSB-PA00367A0Rb, Cusabio), anti-ACC (3662, Cell Signalling), anti-phospho-ACC (3661, Cell Signalling) (all diluted 1:1,000) and anti-vinculin (Sigma, V9131) (diluted 1:2,000). HR-coupled secondary antibodies were from GE Healthcare.

### Analysis of DepMap and Cancer Genome Atlas data

CRISPR (DepMap Public 23Q2+Score, Chronos) and RNAi (Achilles+DRIVE+Marcotte, DEMETER2) essentiality scores for glycolytic enzymes were downloaded from depmap.org. Survival analyses in LIHC were performed using GEPIA2 (ref. ^65^).

### Seahorse analysis

Cells (2 × 10^4^ cells per well) were seeded onto Seahorse XFe96 microplates (Agilent) coated with Cell-Tak (Corning, 354240) using a centrifugation protocol (200*g* for 1 min, zero brake setting). A Glycolysis Stress Test and Mitochondrial Stress Test was performed according to the manufacturer’s protocols. For the Glycolysis Stress Assay, 10 mM glucose was injected, followed by 2 µM oligomycin and 50 mM 2-deoxyglucose. For the Mitochondrial Stress Assay, 2 µM oligomycin was injected, followed by 1 μM FCCP and 0.5 μM rotenone/antimycin A. Energy maps were generated by plotting oxygen consumption rates and extracellular acidification rates before and after addition of oligomycin.

### Metabolomics

For extraction of polar metabolites, a solid phase extraction was used, Cells (~0.5–1 × 10^6^) were washed with cold ammonium acetate (154 mM), snap frozen in liquid nitrogen and scraped off in 0.25 ml ice-cold methanol:H_2_O:acetonitrile (50:20:30 v/v) containing internal standards (MSK-CAA-1, DLM-7654, DLM-9476, DLM-9045, DLM-9071, DLM-6068, DLM-831 and DLM-3487, Cambridge Isotope Laboratories). Samples were subsequently run through a C18 8B-S001-DAK solid phase column (previously activated using acetonitrile and equilibrated using methanol:H_2_O:acetonitrile (50:20:30 v/v)), the eluate was dried in a vacuum concentrator. Dried metabolite extracts were dissolved in 100 μl 5 mM NH_4_OAc in CH_3_CN/H_2_O (75:25, v/v) and 3 µl of each sample was applied to an amide-HILIC column (2.6 μm, 2.1 × 100 mm, Thermo Fisher, 16726-012105). Metabolites were separated at 30 °C by LC using a DIONEX Ultimate 3000 UPLC system and the following solvents: solvent A consisting of 5 mM NH_4_OAc in CH_3_CN:H_2_O (5:95, v/v) and solvent B consisting of 5 mM NH_4_OAc in CH_3_CN:H_2_O (95:5, v/v). The LC gradient programme was: 98% solvent B for 2 min, followed by a linear decrease to 40% solvent B within 5 min, then maintaining 40% solvent B for 13 min, then returning to 98% solvent B in 1 min and then maintaining 98% solvent B for 5 min for column equilibration before each injection. The flow rate was maintained at 350 μl min^−1^. The eluent was directed to the hESI source of the Q Exactive mass spectrometer (QE-MS; Thermo Fisher Scientific) from 1.85 min to 18.0 min after sample injection. The scan range was set to 69.0–1,000 *m*/*z* with a resolution of 70,000 and polarity switching (negative and positive ionization). Peaks corresponding to the calculated metabolites masses taken from an in-house metabolite library (MIM ± 5 ppm) were integrated using the El-MAVEN software v.12.1-beta^66^. For the targeted quantification of FBP, extraction and run were performed as stated above with the following exceptions: 6 µM U-^13^C_6_ FBP (Cambridge Isotope Laboratories, CLM-8962) was used as standard. The LC gradient programme was: 98% solvent B for 2 min, followed by a linear decrease to 30% solvent B within 3 min, then maintaining 30% solvent B for 15 min, then returning to 98% solvent B in 1 min and then maintaining 98% solvent B for 5 min for column equilibration before each injection. The scan range was set to 200–500 *m*/*z* with a resolution of 70,000 and only conducted in negative mode.

### Stable-isotope tracing and glycolytic flux measurements

For glucose and glutamine tracing cells were cultured in MPLM medium containing solvent control (ethanol) or doxycycline (1 μg ml^−1^) for 48 h at which point the cells received fresh MPLM medium for 1 h before being incubated in MPLM medium containing either U-^13^C-glucose (CLM-1396-1) or U-^13^C-glutamine (CLM-1822-H) at 4.4 mM or 1 mM respectively, for 10 h before metabolite extraction and metabolomics analysis. For 1,2-^13^C-glucose tracing, cells first received fresh medium for 1.5 h before incubation in MPLM containing 4.4 mM 1,2-^13^C-glucose (CLM-504-PK Cambridge Isotope Laboratories) for 5, 10, 30 or 240 min before metabolite extraction and metabolomics analysis. Isotopologue distribution of 3PG was determined at isotopic steady state (240 min). Peaks were integrated using the El-MAVEN software and the R package IsoCorrectoR^67^ was used to correct for natural ^13^C abundance. Fractional labelling over time from 1,2-^13^C-glucose tracing was used to determine glycolytic flux. To this end, flux *f* and maximum enrichment *E* were estimated in R using nonlinear fitting of a negative exponential function using the function drc() in the package ‘drc’ and the function DRC.negExp() in the package ‘aomisc’, where *e* is the enrichment at time point *t* (*e* = E(1−*e*^-ft))^68,69^

### Mathematical modelling

To evaluate the relationship between glucose availability, glycolytic flux and ALDOA catalytic activity, we extended a previously published minimal kinetic model of glycolysis^16^. The model consists of five coarse-grained reactions (upper glycolysis, including glucose uptake, aldolase (ALD), lumped lower glycolysis, cellular ATP utilization (ATPase) and provision and utilization of Pi (PiT)) and describes the dynamics of the intracellular concentrations of FBP, triosephosphates (TP), ATP and free Pi using ordinary differential equations.

1 $$\frac{{\rm{d}}\left[{FBP}\right]}{{\rm{d}}t}=+{v}_{{UG}}-{v}_{{ALD}}$$

2 $$\frac{{\rm{d}}\left[{TP}\right]}{{\rm{d}}t}=+2{v}_{{ALD}}-{v}_{{LG}}$$

3 $$\frac{{\rm{d}}\left[{ATP}\right]}{{\rm{d}}t}=-2 {v}_{{UG}}+2 {v}_{{LG}}-{v}_{{ATPase}}$$

4 $$\frac{{\rm{d}}\left[{Pi}\right]}{{\rm{d}}t}=+{v}_{{ATPase}}+{v}_{{PiX}}-2 {v}_{{LG}}$$

All reactions are modelled by kinetic rate equations; parameterization follows van Heerden et al.^16^, with modifications (details of rate equations are described in Supplementary Information). In contrast with van Heerden et al.^16^, the model explicitly describes the dependence of flux on external glucose and expression of aldolase. The model was evaluated with respect to its stability for three different conditions (1) physiological AldoA (100%) and high glycolytic flux ([Glc] = 5 mM); (2) depleted AldoA (20%) and high glycolytic flux ([Glc] = 5 mM); and (3) depleted AldoA (20%) and low glycolytic flux ([Glc] = 1 mM). To test for the robustness of the result with respect to the choice of kinetic parameters, we performed random sampling of parameters (Monte-Carlo analysis), demonstrating that the described instability is an inherent feature of the pathway, independent of the precise choice of parameters. Numerical details are provided in the Supplementary Information.

### Phosphate, ATP, ATP/ADP, NAD(P)^+^/NAD(P)H measurements

Pi was measured using phosphate assay kit (ab65622, Abcam) according to the manufacturer’s instructions, results were then normalized to protein content. Intracellular ATP was measured using a CellTiter-Glo luminescence assay (G7571, Promega) according to the manufacturer’s instructions; results were then normalized to cell density/or protein as assayed on an identical plate plated in parallel using crystal violet or BCA assay. The ATP:ADP ratio was measured using an ADP:ATP ratio assay kit (MAK135, Sigma) according to the manufacturer’s instructions. The NAD(P)^+^:NAD(P)H ratio was measured using NAD:NADH-Glo and NADP:NADPH-Glo assays (G9071 and G9081, Promega) according to the manufacturer’s instructions.

### Oxidative stress measurements

Cells were cultured in medium containing 2 µM CellROX deep red reagent (C10491 Molecular Probes) and red fluorescence was monitored every 2 h using an Incucyte live-cell imaging instrument (Sartorius). As a positive control, cells were treated with 100 µM tert-butyl hydroperoxide (C10491 Molecular Probes).

### Flow cytometry

Cells were treated with 10 µM bromodeoxyuridine (BrdU) for 30 min before being collected by trypsinization. The pellet was washed with cold PBS and fixed in 80% ethanol. Subsequently, 500,000 cells were washed in PBS and incubated for 30 min in 2 M HCl/0.5% Triton X-100 at room temperature. Cells were centrifuged at 400*g*, resuspended in 0.1 M Na2B4O7 (pH8.5) centrifuged again at 400*g* and then incubated for 30 min in 100 μl 1% BSA in PBS-T + 10 μl anti-BrdU V450 (BD, 560810) antibody. The cells were then washed in 1% BSA in PBS-T before being incubated in 38 mM citrate solution containing propidium iodide at a concentration of 54 µM and then analysed on a BD FACS-Canto II flow cytometer using BD FACSDIVA software. Cell cycle analysis was performed using FlowJo (v.10).

### Animal experiments

All animal experiments were approved by the committees of the regional authority of the state of Bavaria (Regierungspräsidium Unterfranken, RUF 55.2.2-2532-2-751) and the state of Baden-Württemberg (Regierungspräsidium Karlsruhe, G-107/20). All mice were housed and maintained under pathogen-free conditions with a 12-h light–dark cycle, 45–65% relative humidity, temperature of 22 ± 2 °C and ad libitum access to water and food in accordance with institutional guidelines. Hydrodynamic tail vein injection was performed in 6–7-week-old male or female C57BL/6N WT mice. Pilot experiments confirmed no significant difference between survival of male and female mice in this model. Mice were randomly allocated to the different groups and injected with 25 µg of transposon plasmids (*pT-CaMIA-shRen*, *pT-CaMIA-shAldoa.1280* or *pT-CaMIA-shAldoa.558*) together with 5 µg of plasmid coding for the Sleeping Beauty (SB) transposase diluted in 0.9% NaCl solution to a final volume of 10% of mouse body weight. DNA for hydrodynamic tail vein injection was generated using the QIAGEN EndoFreeMaxi kit. Animals that were not efficiently injected within 10 s were excluded from the experiments. Mice were observed until the predefined humane end point had been reached and differences in survival were determined by Kaplan–Meier analysis using a log-rank test. The SB transposase (pSB13) and the *pT-CaMIA* transposon plasmids have been described previously^27,70^. The shRNAs were shuttled into *pT-CaMIA* using XhoI and MluI–AscI fragments.

### Immunohistochemistry

Paraffin-embedded sections of murine liver samples were cut into 3-µm sections with a microtome (HM 355S). Slides were deparaffinized, hydrated and submitted to antigen retrieval and blocking. Slides were stained with anti-Ki67 (IHC-00375, 1:300 dilution, Bethyl Laboratories), developed with Alkaline Phosphatase Streptavidin (SA-5100, 1:200 dilution, Vector Laboratories) and counterstained with haematoxylin (Carl Roth, T865.3). Slides were scanned at ×20 resolution using a slide scanner (Zeiss Axioscan 7) and analysed using QuPath (v.0.3.2).

### Statistical analysis

Statistical analyses of data obtained from metabolomics were performed with MetaboAnalyst v.6.0 (www.metaboanalyst.ca). Statistical analyses of the other data were carried out with GraphPad Prism v.9. Groups were compared by multivariate (principal-component analysis) and/or univariate analyses (*t*-test or analysis of variance (ANOVA), *P* < 0.05, false discovery rate (FDR)-applied) followed by post hoc tests, unless otherwise stated in the figure legend. No statistical methods were used to predetermine sample sizes for cell culture experiments. Power analysis was performed to determine size of animal cohorts based on pilot experiments (type 1 error of 5%; type 2 error of 20%; Software R, v.4.3.0). Data distribution was assumed to be normal but this was not formally tested. Randomization was performed for animal experiments. Image analysis of tissue samples was performed in a blinded manner using automated methods. No animals or data points were excluded. All data were presented as mean ± s.d. of independent biological replicates with the number of replicates stated in the figure legends.

### Reporting summary

Further information on research design is available in the Nature Portfolio Reporting Summary linked to this article.

## Supplementary information

Supplementary Information Supplementary Modelling and Supplementary Figures.

Reporting Summary

Supplementary Table 1 Sequences of shRNA library used for dropout screen.

Supplementary Table 2 Results of dropout screen as MAGeCK essentiality scores.

Supplementary Table 3 Guide RNA sequences used in this study.

Supplementary Table 4 Composition of the mouse plasma-like medium used in this study.

## Source data

Source Data Fig. 2 Statistical source data.

Source Data Fig. 3 Statistical source data.

Source Data Fig. 4 Statistical source data.

Source Data Fig. 5 Statistical source data.

Source Data Fig. 6 Statistical source data.

Source Data Fig. 7 statistical source data.

Source Data Fig. 8 Statistical source data.

Source Data Fig. 8 Gating strategy.

Source Data Original immunoblots.

Source Data Extended Data Original immunoblots.

Source Data Extended Data Fig. 1 Statistical source data.

Source Data Extended Data Fig. 2 Statistical source data.

Source Data Extended Data Fig. 3 Statistical source data.

Source Data Extended Data Fig. 4 Statistical source data.

Source Data Extended Data Fig. 5 Statistical source data.

Source Data Extended Data Fig. 6 Statistical source data.

Source Data Extended Data Fig. 7 Statistical source data.

Source Data Extended Data Fig. 8 Statistical source data.

## Data Availability

Source data are provided as supplementary materials. All other data and materials will be made available upon request. Source data are provided with this paper.
