# Supplementary material for: Targeting aldolase A in hepatocellular carcinoma leads to imbalanced glycolysis and energy stress due to uncontrolled FBP accumulation
Source: Nat Metab. 2025 Jan 20;7(2):348–66. doi: 10.1038/s42255-024-01201-w (PMC11860237; doi:10.1038/s42255-024-01201-w)
Supplement: Supplementary file 1 — Supplementary Modelling and Supplementary Figures. [file 42255_2024_1201_MOESM1_ESM.pdf]

nature metabolism

Supplementary information https://doi.org/10.1038/s42255-024-01201-w

Targeting aldolase A in hepatocellular carcinoma leads to imbalanced glycolysis and energy stress due to uncontrolled FBP accumulation

In the format provided by the authors and unedited

SUPPLEMENTARY INFORMATION: MODELLING

TARGETING ALDOLASE A IN HEPATOCELLULAR CARCINOMA LEADS TO IMBALANCED GLYCOLYSIS AND ENERGY STRESS DUE TO FBP ACCUMULATION

SNAEBJORNSSON ET AL. (2024)

MODEL DEFINITIONS

The coarse-grained kinetic model of glycolysis is based on a model previously described by van Heerden et al. (2014) and modified according to the requirements of our study. The model consists of 5 biochemical reactions: upper glycolysis (UG), aldolase (ALD), lower glycolysis (LG), ATP utilization (ATPase), as well as exchange of inorganic phosphate (PiX),

$$\mathrm{GLC_e} + 2\,\mathrm{ATP} \rightarrow 2\,\mathrm{ADP} + \mathrm{FBP} \quad (1)$$

$$\mathrm{FBP} \leftrightarrow 2\,\mathrm{TP} \quad (2)$$

$$\mathrm{TP} + 2\,\mathrm{ADP} + \mathrm{Pi} \rightarrow 2\,\mathrm{ATP} + \mathrm{PYR_e} \quad (3)$$

$$\mathrm{ATP} \rightarrow \mathrm{Pi} + \mathrm{ADP} \quad (4)$$

$$\mathrm{Pi_e} \leftrightarrow \mathrm{Pi}\,. \quad (5)$$

The model describes the dynamics of the intracellular concentrations of FBP, triosephosphates (TP), ATP, and free inorganic phosphate (Pi). The model includes a conserved moiety $\sum_A$ = [ATP] + [ADP] (total adenylate). The concentrations of the independent intracellular variables are described by ordinary differential equations (ODEs),

$$\frac{\mathrm{d[FBP]}}{\mathrm{d}t} = +v_{UG} - v_{ALD} \quad (6)$$

$$\frac{\mathrm{d[TP]}}{\mathrm{d}t} = +2 \cdot v_{ALD} - v_{LG} \quad (7)$$

$$\frac{\mathrm{d[ATP]}}{\mathrm{d}t} = -2 \cdot v_{UG} + 2 \cdot v_{LG} - v_{ATPase} \quad (8)$$

$$\frac{\mathrm{d[Pi]}}{\mathrm{d}t} = +v_{ATPase} + v_{PiX} - v_{LG} \quad . \quad (9)$$

A schematic representation is provided as Fig. S1.

All reactions are described using kinetic rate equations. The rate of upper glycolysis depends on the availability of extracellular glucose (GLC_e), the expression of glucose transporter, and all reactions of upper glycolysis, including glucose 6-phosphate isomerase (GPI). The maximal reaction velocities of the respective reactions are lumped into a single maximal reaction velocity for upper glycolysis $v_{max,UG}$. Following Teusink et al. (2000), the aldolase reaction is described by an ordered uni-bi rate equation. Lower glycolysis describes the interconversion of TP to the final product, intracellular pyruvate (PYR_e). ATP utilization is described by irreversible mass action kinetics. Finally, and similar to van Heerden et al. (2014), we assume

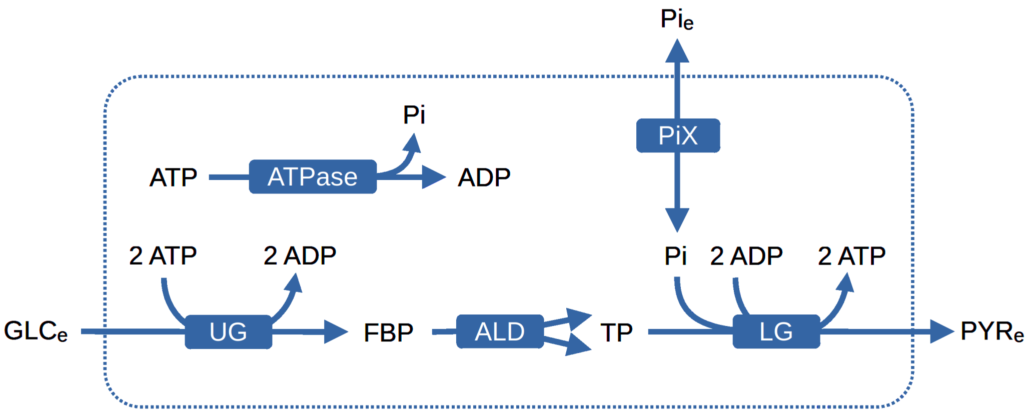

Figure S1: A schematic representation of the coarse-grained kinetic model of glycolysis. The model consists of 5 biochemical reactions (upper glycolysis, UG; aldolase A, ALD; lower glycolysis, LG; ATP utilization, ATPase; phosphate exchange, PiX) and 5 intracellular metabolite concentrations whose dynamics are described by 4 ordinary differential equations (ODEs) and the mass conservation relationship $\sum_A = [\mathrm{ATP}] + [\mathrm{ADP}]$.

exchange of Pi within the cell. Pi exchange is described by an influx and withdrawal of Pi (lumped into a single reversible reaction). At steady state both fluxes are balanced. The model makes no assumption whether the influx is due to intracellular mobilization of Pi or due to import from the medium.

The definition of the ODEs requires 19 kinetic parameters: 3 maximal reaction velocities (for UG, ALD, LG), 2 rate constants for mass-action kinetics (ATPase and PiX), 10 Michaelis-Menten (or affinity) constants, a thermodynamic equilibrium constant for ALD reaction, the total adenylate moiety $\sum_A$, a parameter for Pi influx, and the concentration $[\mathrm{GLC}_e]$ of external glucose. All rate equations and numerical values for parameters are provided below.

We emphasize that our aim is not a detailed description of the biochemistry, but to demonstrate that the experimentally observed behavior is a consequence of the pathway topology and can be recapitulated for a broad range of kinetic parameters. Specifically, the model demonstrates that (1) reducing aldolase activity can trigger an imbalanced glycolytic state where FBP accumulates continuously, causing depletion of Pi and ATP, (2) the imbalanced state can be rescued by either a decrease in external glucose concentration or a decrease in the maximal activity of upper glycolysis.

TIME COURSE SIMULATIONS

Simulation were conducted using the rate equations and kinetic parameters described below, the results are shown as Fig. 5d in the main text. The dynamics are characterized by a fast initial transient to the steady state. For imbalanced glycolysis (Fig. 5d, middle graph), the fast transient dynamics (depletion of ATP and Pi) are followed by a slow increase in the concentration of FBP. The increase is determined by the rate of the phosphate (Pi) exchange reaction (PiX),

the respective parameter are chosen to match Fig. 5b in the main text. Simulations were performed using a Python binding for CVODES integrator (https://pypi.org/project/pycvodes/). For timecourses shown in Fig. 5d left and middle graph, the initial conditions were: Pi at 8.0 mmol/l, ATP at 1.0 mmol/l, ADP at 4.0 mmol/l, FBP at 2.0 mmol/l, and TP at 1.5 mmol/l. For the right graph, the initial conditions were Pi at 0.8 mmol/l, ATP at 0.3 mmol/l, ADP at 4.7 mmol/l, FBP at 4.9 mmol/l, and TP at 0 mmol/l. Initial conditions have no impact on the dynamics of the model after the initial transient.

Bifurcation Analysis

To demonstrate the transition into an imbalanced glycolytic state for a wider range of values for external glucose and AldoA expression, we conducted a bifurcation analysis with respect to the stability of the steady-state. To this end, the concentration of external glucose ([GLC_e]) and the maximal reaction velocity of AldoA catalysis ($v_{max,ALD}$) were systematically varied. The results are depicted in Fig. 5g of the main text and show that the transition into the unstable state can be due to a reduction of the maximum reaction velocity of AldoA (AldoA activity) or by an increase of the concentration of external glucose. Within the coarse-grained model, a reduction of glucose availability and a reduction of GPI have similar effects.

Monte-Carlo Analysis

To further identify the conditions that give rise to imbalanced glycolysis, we employed a probabilistic approach that allows us to assess the stability of the metabolic state independent of the choice of kinetic parameters. To this end, we follow the approach described by Murabito et al. (2014) [*Monte-Carlo Modeling of the Central Carbon Metabolism of Lactococcus lactis: Insights into Metabolic Regulation.* PLoS ONE 9(9): e106453] to construct an ensemble of models that give rise to an identical steady-state, but differ in their respective kinetic parameters.

In brief, within each iteration, all Michaelis-Menten parameters are chosen randomly in a defined interval and the reaction velocities are chosen such that the model gives rise to the defined metabolic state, characterized by the steady-state fluxes and concentrations (x^0, v^0), see Murabito et al. (2014) for computational details. Subsequently the partial derivatives of the reaction rates are calculated. The partial derivatives quantify how much a change in a concentration x changes a reaction rate v, and are known as *scaled elasticity coefficients* in the context of metabolic control analysis (MCA),

$$\hat{\epsilon}_x^v = \frac{x^0}{v^0} \frac{\partial v}{\partial x}\bigg|_{x^0} = \frac{\partial \ln v}{\partial \ln x}\bigg|_{x^0} \quad . \tag{10}$$

The scaled elasticities are evaluated at the given steady state (x^0, v^0) and describe the (inverse of the) "degree of saturation" of a reaction with respect to the concentration of the metabolite x. That is, a scaled elasticity $\hat{\epsilon}_x^v \approx 0$ implies that the reaction is fully saturated with respect to its substrate x, whereas a value $\hat{\epsilon}_x^v \approx 1$ implies a linear dependence of the reaction rate on its substrate x.

Finally, knowledge of the partial derivatives allows us to calculate the Jacobian matrix. The stability of the steady state is determined by the largest real part of the eigenvalues of the Jacobian.

Using the stable steady state from the original parameterization as a reference,

Steady state concentrations, mmol/l				
[ATP]	[ADP]	[FBP]	[TP]	[Pi]
1 . 04	3 . 96	1 . 61	0 . 29	10

Steady state fluxes, mmol/l/min				
v_{UG}	v_{ALD}	v_{LG}	v_{ATPase}	v_{PiX}
4.16	4.16	8.32	8.32	0

we generated an ensemble of models by choosing all Michaelis-Menten parameters (including inhibition constants) randomly using a logarithmic distribution around its reference value K_M^{ref}, such that $K_M \in [10^{-n} \cdot K_M^{ref}, 10^{+n} \cdot K_M^{ref}]$. In the following $n = 2$. For each set of sampled K_M values, the stability of the model was evaluated by calculating the largest real part of the eigenvalues of the Jacobian.

Using the calculated values for the scaled elasticities, we can determine the conditions under which the steady state becomes unstable. Figure S2 shows the sampled elasticities, such that each dot corresponds to a (random) realization of the model. Dots are color-coded to indicate whether the steady-state is stable or unstable. The figure shows that a main determinant of stability is the degree of saturation (the scaled elasticity) of lower glycolysis with respect to Pi, relative to the degree of saturation of lower glycolysis with respect to TP. Specifically, depletion of free Pi increases the value of the scaled elasticity of lower glycolysis with respect to Pi, thereby increasing the possibility of imbalanced glycolysis. Decreasing the Aldolase increasingly depletes free Pi as Pi become sequestered in the metabolites (specifically FBP) of upper glycolysis.

Kinetic rate equations and parameters

The definition of rate laws follows van Heerden et al. (2014) unless otherwise noted. Parameters are chosen according to (1) van Heerden et al. (2014), (2) Teusink et al. (2000), or (3) defined in this study.

Upper glycolysis (UG)

$$\mathrm{GLC_e} + 2\,\mathrm{ATP} \rightarrow 2\,\mathrm{ADP} + \mathrm{FBP}$$

$$v_{UG} = \frac{v_{max,UG} \cdot [\mathrm{ATP}]}{K_{m,PFK,ATP} + [\mathrm{ATP}] \cdot \left(1 + \frac{[\mathrm{ATP}]}{K_{i,PFK,ATP}}\right)} \cdot \frac{[\mathrm{GLC_e}]}{[\mathrm{GLC_e}] + K_{m,GLC}}$$

Parameter	Value	Unit	Source
$v_{max,UG}$	12	mmol/l/min	(3)
$K_{m,PFK,ATP}$	0.1	mmol/l	(1)
$K_{i,PFK,ATP}$	3	mmol/l	(1)
$K_{m,GLC}$	5	mmol/l	(3)
$[GLC_e]$	5	mmol/l	(3)

ALDOLASE (ALD)

$$\mathrm{FBP} \leftrightarrow 2\ \mathrm{TP}$$

$$v_{ALD} = v_{max,ALD} \cdot \left([\mathrm{FBP}] - \frac{[\mathrm{TP}]^2}{k_{eq,ALD}} \right) \cdot \frac{1}{D}$$

$$D = K_{m,ALD,FBP} \cdot (1 + \frac{[\mathrm{FBP}]}{K_{m,ALD,FBP}} + \frac{[\mathrm{TP}]}{K_{m,ALD,DHAP}} + \frac{[\mathrm{TP}]}{K_{m,ALD,GAP}} + \\ + \frac{[\mathrm{FBP}] \cdot [\mathrm{TP}]}{K_{m,ALD,FBP} \cdot K_{i,ALD,GAP}} + \frac{[\mathrm{TP}]^2}{K_{m,ALD,DHAP} \cdot K_{m,ALD,GAP}})$$

Parameter	Value	Unit	Source
$v_{max,ALD}$	24	mmol/l/min	(3)
$k_{eq,ALD}$	0.069	1	(2)
$K_{m,ALD,DHAP}$	2.4	mmol/l	(2)
$K_{m,ALD,GAP}$	2	mmol/l	(2)
$K_{m,ALD,FBP}$	0.3	mmol/l	(2)
$K_{i,ALD,GAP}$	10	mmol/l	(2)

LOWER GLYCOLYSIS (LG)

$$\mathrm{TP} + 2\ \mathrm{ADP} + \mathrm{Pi} \rightarrow 2\ \mathrm{ATP} + \mathrm{PYR_e}$$

$$v_{LG} = v_{max,LG} \cdot \frac{[\mathrm{TP}]}{K_{m,LG,TP} + [\mathrm{TP}]} \cdot \frac{[\mathrm{ADP}]}{K_{m,LG,ADP} + [\mathrm{ADP}]} \cdot \frac{[\mathrm{Pi}]}{K_{m,LG,Pi} + [\mathrm{Pi}]}$$

Parameter	Value	Unit	Source
$v_{max,LG}$	45	mmol/l/min	(3)
$K_{m,LG,TP}$	1	mmol/l	(1)
$K_{m,LG,ADP}$	0.1	mmol/l	(1)
$K_{m,LG,Pi}$	2	mmol/l	(1)

ATPASE

$$\mathrm{ATP} \rightarrow \mathrm{Pi} + \mathrm{ADP}$$

$$v_{ATPase} = k_{ATPase} \cdot [\mathrm{ATP}]$$

Parameter	Value	Unit	Source
k_{ATPase}	8	1/min	(3)
$\sum_A$	5	mmol/l	(1)

PHOSPHATE EXCHANGE (PIX)

$$\mathrm{Pi_e} \leftrightarrow \mathrm{Pi}$$

$$v_{PiX} = k_{PiX} \cdot ([\mathrm{Pi_e}] - [\mathrm{Pi}])$$

Parameter	Value	Unit	Source
k_{PiX}	0.005	1/min	(3)
$[\mathrm{Pi}_e]$	10	mmol/l	(1)

ADDITIONAL REFERENCES

- Murabito et al. (2014) Monte-Carlo Modeling of the Central Carbon Metabolism of Lactococcus lactis: Insights into Metabolic Regulation. PLoS ONE 9(9): e106453
- Teusink et al. (2000) Can yeast glycolysis be understood in terms of in vitro kinetics of the constituent enzymes? Testing biochemistry. Eur J Biochem. 267(17):5313-29. doi: 10.1046/j.1432-1327.2000.01527.x.

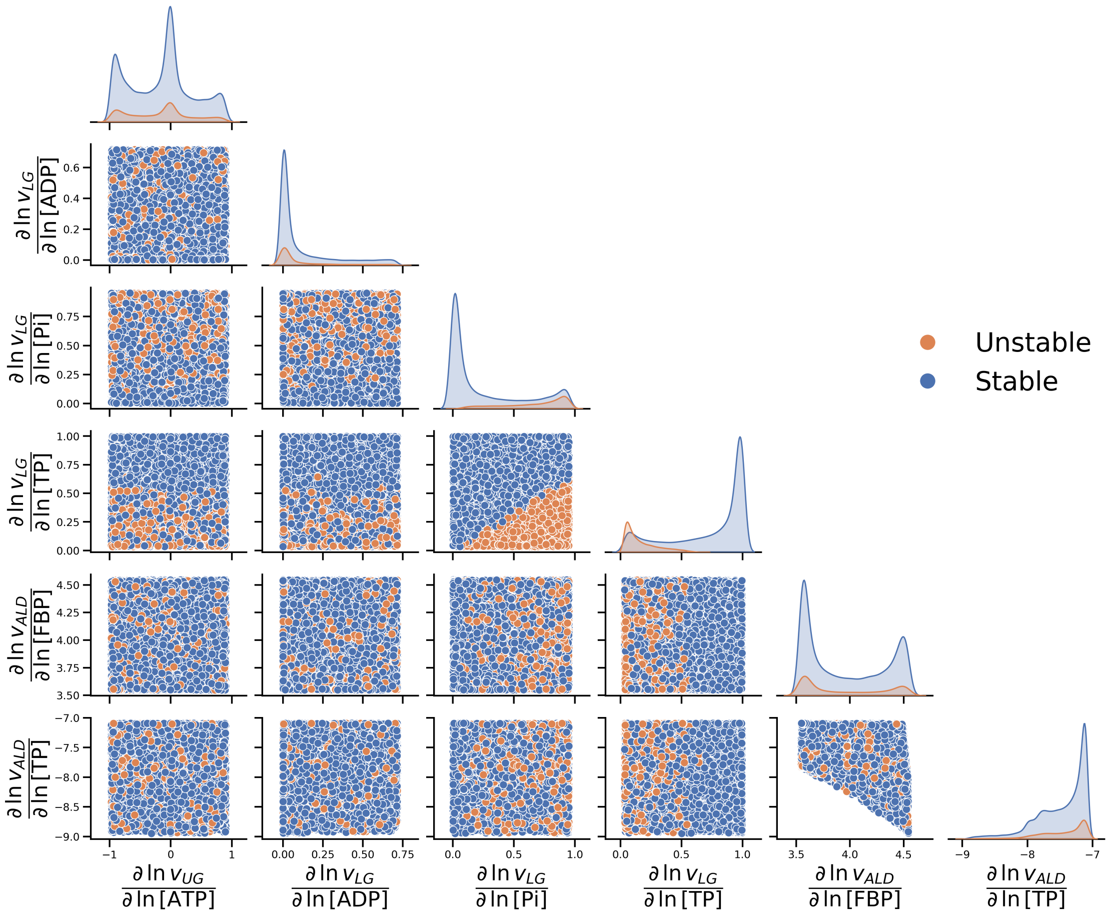

Figure S2: Monte-Carlo analysis of the stability of the coarse-grained kinetic model of glycolysis. Each dot corresponds to a random instance of the model with different (randomly sampled) Michaels-Menten parameters. For each realization, the scaled elasticities were calculated (the respective distributions are shown on the diagonal). Dots are color-coded whether the respective model instance is stable or unstable. Shown are 10^5 random realizations of the model, 83.72% of all realizations exhibit a stable steady state. Stability is primarily associated with the scaled elasticities of lower glycolysis with respect to its substrates Pi and TP. Density estimation was performed using the function `gaussian_kde` from the `scipy.stats` module in Python.
