# Supplementary material for: Targeting aldolase A in hepatocellular carcinoma leads to imbalanced glycolysis and energy stress due to uncontrolled FBP accumulation
Source: Nat Metab. 2025 Jan 20;7(2):348–66. doi: 10.1038/s42255-024-01201-w (PMC11860237; doi:10.1038/s42255-024-01201-w)
Supplement: Supplementary file 2 — Reporting Summary [file 42255_2024_1201_MOESM2_ESM.pdf]

nature portfolio

Corresponding author(s): Almut Schulze

Last updated by author(s): 03/12/2024

Reporting Summary

Nature Portfolio wishes to improve the reproducibility of the work that we publish. This form provides structure for consistency and transparency in reporting. For further information on Nature Portfolio policies, see our Editorial Policies and the Editorial Policy Checklist.

Statistics

For all statistical analyses, confirm that the following items are present in the figure legend, table legend, main text, or Methods section.

n/a	Confirmed	
☐	☒	The exact sample size (*n*) for each experimental group/condition, given as a discrete number and unit of measurement
☐	☒	A statement on whether measurements were taken from distinct samples or whether the same sample was measured repeatedly
☐	☒	The statistical test(s) used AND whether they are one- or two-sided<br>*Only common tests should be described solely by name; describe more complex techniques in the Methods section.*
☒	☐	A description of all covariates tested
☐	☒	A description of any assumptions or corrections, such as tests of normality and adjustment for multiple comparisons
☐	☒	A full description of the statistical parameters including central tendency (e.g. means) or other basic estimates (e.g. regression coefficient) AND variation (e.g. standard deviation) or associated estimates of uncertainty (e.g. confidence intervals)
☐	☒	For null hypothesis testing, the test statistic (e.g. *F*, *t*, *r*) with confidence intervals, effect sizes, degrees of freedom and *P* value noted<br>*Give P values as exact values whenever suitable.*
☐	☒	For Bayesian analysis, information on the choice of priors and Markov chain Monte Carlo settings
☒	☐	For hierarchical and complex designs, identification of the appropriate level for tests and full reporting of outcomes
☒	☐	Estimates of effect sizes (e.g. Cohen's *d*, Pearson's *r*), indicating how they were calculated

Our web collection on statistics for biologists contains articles on many of the points above.

Software and code

Policy information about availability of computer code

Data collection	N/A
Data analysis	Software used for data analysis was: FlowJo (version 10), Graphpad Prism 9, MetaboAnalyst 6.0, El-MAVEN v.12.1-beta, R package IsoCorrectoR<br>Software tools used for analysis of public data was: Depmap (https://depmap.org/portal/), GEPIA2 (http://gepia2.cancer-pku.cn/#index). .<br>All code used for modeling is provided as supplementary information

For manuscripts utilizing custom algorithms or software that are central to the research but not yet described in published literature, software must be made available to editors and reviewers. We strongly encourage code deposition in a community repository (e.g. GitHub). See the Nature Portfolio guidelines for submitting code & software for further information.

Data

Policy information about availability of data

All manuscripts must include a data availability statement. This statement should provide the following information, where applicable:

- Accession codes, unique identifiers, or web links for publicly available datasets
- A description of any restrictions on data availability
- For clinical datasets or third party data, please ensure that the statement adheres to our policy

Publicly available datasets were from TCGA and analyzed using GEPIA2 (http://gepia2.cancer-pku.cn/#index). Raw data are provided as supplementary information.

A statement regarding data availability is included in the manuscript. "Source data are provided as supplementary material. All other data and material will be made available upon request.".

Research involving human participants, their data, or biological material

Policy information about studies with human participants or human data. See also policy information about sex, gender (identity/presentation), and sexual orientation and race, ethnicity and racism.

Reporting on sex and gender	N/A
Reporting on race, ethnicity, or other socially relevant groupings	N/A
Population characteristics	N/A
Recruitment	N/A
Ethics oversight	N/A

Note that full information on the approval of the study protocol must also be provided in the manuscript.

Field-specific reporting

Please select the one below that is the best fit for your research. If you are not sure, read the appropriate sections before making your selection.

☒ Life sciences ☐ Behavioural & social sciences ☐ Ecological, evolutionary & environmental sciences

For a reference copy of the document with all sections, see nature.com/documents/nr-reporting-summary-flat.pdf

Life sciences study design

All studies must disclose on these points even when the disclosure is negative.

Sample size	Sample size is provided in the figure legends. No statistical methods were used to pre-determine sample sizes for cell culture experiments. Power analysis was performed to determine size of animal cohorts based on pilot experiments (type 1 error = 5%; type 2 error = 20%; Software R, Version 4.3.0). Data distribution was assumed to be normal but this was not formally tested.
Data exclusions	No data were excluded
Replication	Numbers of biologically independent replicate samples are provided in the figure legend. All data are derived from at least three biological replicates.
Randomization	Mice were randomised into different treatment groups. No randimisation was carried out for in vitro experiments.
Blinding	Investigators were not blinded for the analysis of the data derived from tissue culture experiments. Analysis of histological samples was performed in a blinded manner. Most of the key findings of the study were confirmed several times by different investigators. Data were analyzed using established pipelines and independently checked by several investigators.

Reporting for specific materials, systems and methods

We require information from authors about some types of materials, experimental systems and methods used in many studies. Here, indicate whether each material, system or method listed is relevant to your study. If you are not sure if a list item applies to your research, read the appropriate section before selecting a response.

Materials & experimental systems

n/a	Involved in the study	
☐	☒	Antibodies
☐	☒	Eukaryotic cell lines
☒	☐	Palaeontology and archaeology
☐	☒	Animals and other organisms
☒	☐	Clinical data
☒	☐	Dual use research of concern
☒	☐	Plants

Methods

n/a	Involved in the study	
☒	☐	ChIP-seq
☐	☒	Flow cytometry
☒	☐	MRI-based neuroimaging

Antibodies

Antibodies used	Providers of commercially available antibodies used in this study are provided in the Methods section. Antibodies used:<br>Western blotting: anti-ALDOA (11217-1-AP Proteintech or H00000226-M01 Novus), anti-GPI (15171-1-AP Proteintech or CSB-PA00367A0Rb Cusabio), anti-ACC (3662 Cell Signalling), anti-phospho-ACC (3661 Cell Signalling) all used at 1:1000; anti-Vinculin (Sigma, V9131) used at 1:2000.<br>Histology: anti-Ki67 (IHC-00375, Bethyl Laboratories) used at 1:300.
Validation	For Western Blotting: All antibodies were obtained from trusted sources and were confirmed to detect proteins of the correct size.<br>For anti-AldoA (11217-1-AP Proteintech) and anti-Gpi (15171-1-AP Proteintech or CSB-PA00367A0Rb Cusabio), gene silencing was used to validate the identify of the detected bands of the correct size.<br>For anti-ACC (3662 Cell Signaling) and anti-phospho-ACC (3661 Cell Signaling). The vendor cites 707 publications using anti-ACC and 1177 citations using anti-phsopho-ACC. In addition, control experiments using glucose starvation to activate AMPK were performed.<br>For anti-Vinculin (Sigma, V9131), the vendor states "independent antibody verification" (different antibodies show similar experimental results).<br>For Histology: anti-Ki67 antibody (IHC-00375, Bethyl Laboratories) is routinely used by the DKFZ Histology Core Facility.

Eukaryotic cell lines

Policy information about cell lines and Sex and Gender in Research

Cell line source(s)	Murine liver cancer cell lines were directly derived from animal models and obtained from D. Dauch (Tübingen). Human liver cancer cell lines were originally from ATCC and kindly shared by P. Friedmann Angeli (Würzburg).
Authentication	All cell lines were obtained from a trusted source and used at low passage. No STR profiling was carried out for human cell lines. STR profiling is not possible for the primary mouse cell lines used for the majority of in vitro experiments.
Mycoplasma contamination	All cell lines were regularly tested for the absence of mycoplasma contamination and found to be negative.
Commonly misidentified lines<br>(See ICLAC register)	None of the commonly misidentified cell lines listed in the ICLAC register have been used in our study.

Animals and other research organisms

Policy information about studies involving animals; ARRIVE guidelines recommended for reporting animal research, and Sex and Gender in Research

Laboratory animals	C57Bl/6 N were used for hydrodynamic tail vein injection at an age of 6 to 7 weeks.
Wild animals	No wild animals were used in our study.
Reporting on sex	Both male and female (approx. equal numbers) were used
Field-collected samples	No field collected samples were used in our study.
Ethics oversight	All animal experiments have been approved by committees of the regional authority of the state of Bavaria (Regierungspräsidium Unterfranken, RUF 55.2.2-2532-2-751) and the state of Baden-Württemberg (Regierungspräsidium Karlsruhe, G-107/20). All mice were housed and maintained under pathogen free conditions in accordance with the institutional guidelines.

Note that full information on the approval of the study protocol must also be provided in the manuscript.

Plants

Seed stocks	N/A
Novel plant genotypes	N/A
Authentication	N/A

Flow Cytometry

Plots

Confirm that:

☒ The axis labels state the marker and fluorochrome used (e.g. CD4-FITC).

☒ The axis scales are clearly visible. Include numbers along axes only for bottom left plot of group (a 'group' is an analysis of identical markers).

☒ All plots are contour plots with outliers or pseudocolor plots.

☒ A numerical value for number of cells or percentage (with statistics) is provided.

Methodology

Sample preparation	Provided in the Methods
Instrument	Provided in the Methods
Software	Provided in the Methods
Cell population abundance	Provided in supplementary information.
Gating strategy	Provided in supplementary information.

☒ Tick this box to confirm that a figure exemplifying the gating strategy is provided in the Supplementary Information.
