# Supplementary figures and images for: Targeting aldolase A in hepatocellular carcinoma leads to imbalanced glycolysis and energy stress due to uncontrolled FBP accumulation
Source: Nat Metab. 2025 Jan 20;7(2):348–66. doi: 10.1038/s42255-024-01201-w (PMC11860237; doi:10.1038/s42255-024-01201-w)

# Gating strategy for FACS

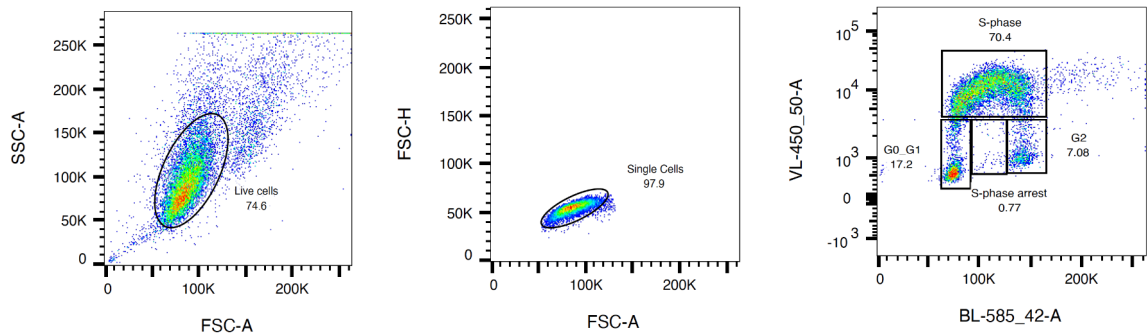

Supplement: Supplementary file 14 — Gating strategy. [file 42255_2024_1201_MOESM14_ESM.pdf]

Figure 2c

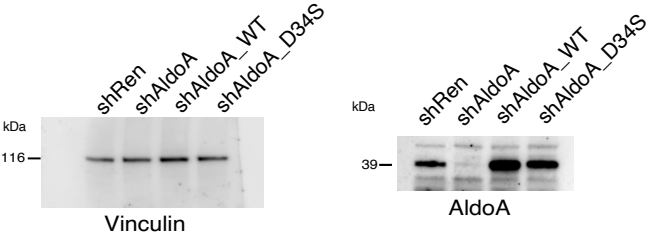

Figure 3b

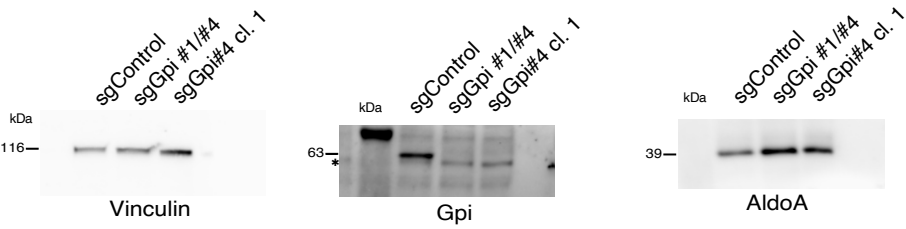

Figure 4g

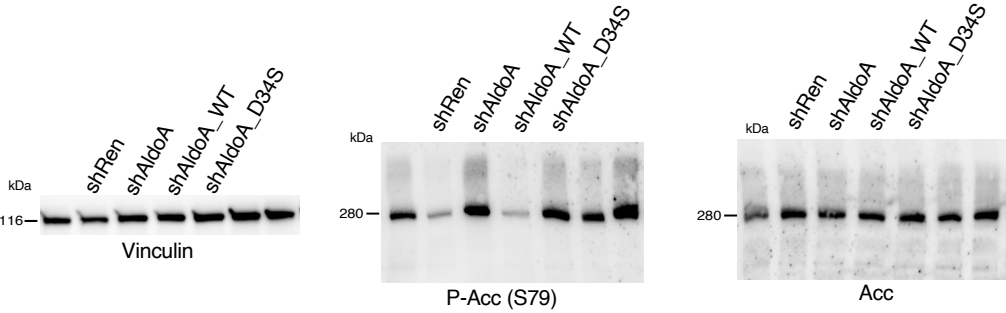

Figure 5i

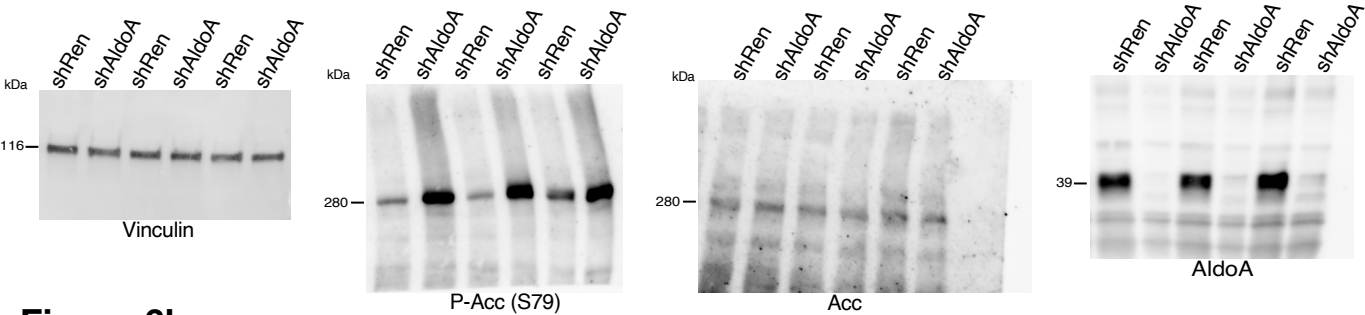

Figure 6b

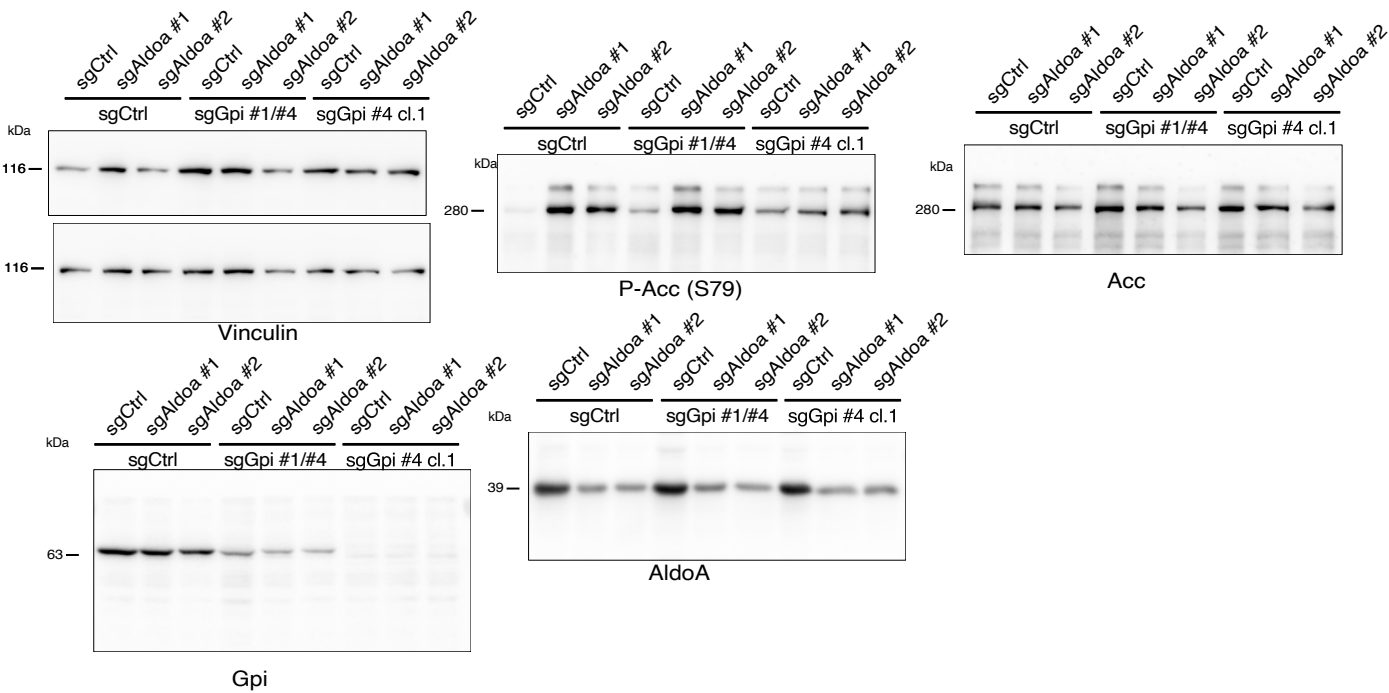

Supplement: Supplementary file 15 — Original immunoblots. [file 42255_2024_1201_MOESM15_ESM.pdf]

Figure S2b

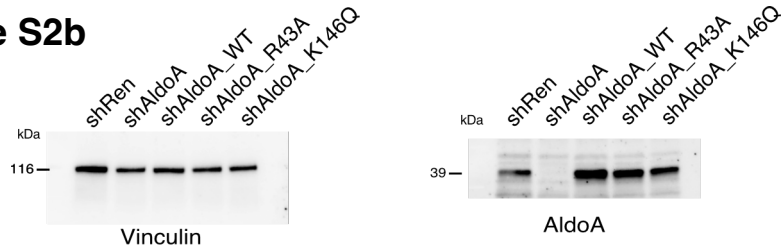

Figure S3b

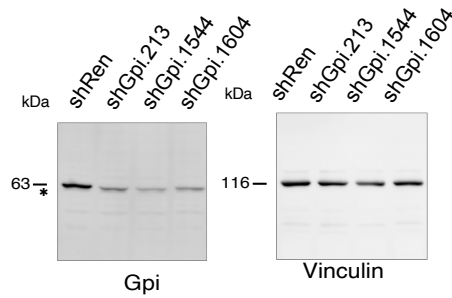

Figure S3d

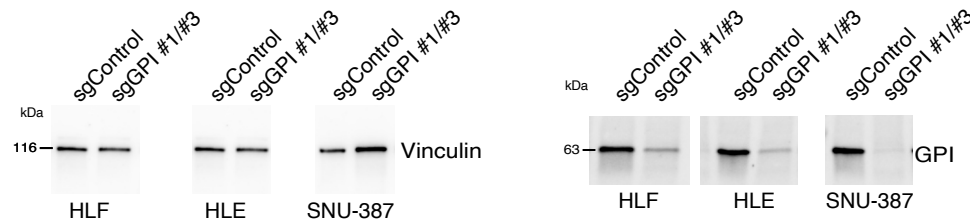

Figure S6c

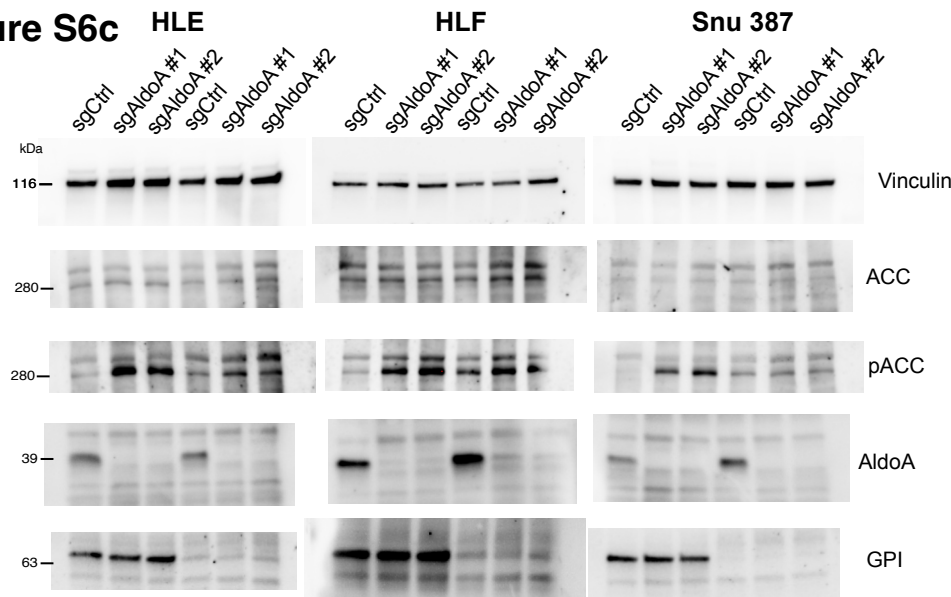

Supplement: Supplementary file 16 — Original immunoblots. [file 42255_2024_1201_MOESM16_ESM.pdf]
